# 72nd Congress of the Italian Society of Pediatrics: Florence, Italy. 16-19 November 2016

**DOI:** 10.1186/s13052-017-0327-2

**Published:** 2017-02-20

**Authors:** Marco Braghero, Annamaria Staiano, Eleonora Biasin, Patrizia Matarazzo, Silvia Einaudi, Rosaria Manicone, Francesco Felicetti, Enrico Brignardello, Franca Fagioli, Elisabetta Bignamini, Elena Nave, F. Callea, C. Concato, E. Fiscarelli, S. Garrone, M.Rossi de Gasperis, Patrizia Calzi, Grazia Marinelli, Roberto Besana, Carlo Caffarelli, Antonio Di Peri, Irene Lapetina, Patrizia Cincinnati, Rosalia Maria Da Riol, Mario De Curtis, Lucia Dito, Chiara Protano, Susanna Esposito, Dante Ferrara, Rossella Galiano, Pasquale Novellino, Eric Heath Kossoff, Andrzej Krzysztofiak, Elena Bozzola, Laura Lancella, Alessandra Marchesi, Alberto Villani, Paola Lago, Elisabetta Garetti, Anna Pirelli, Paola Marchisio, Maria Santagati, Stefania Stefani, Susanna Esposito, Nicola Principi, Valeria d’Apolito, Luigi Memo, Angelo Selicorni, Vito Leonardo Miniello, Lucia Diaferio, Antonella Palmieri, Luciana Parola, Ettore Piro, Claudio Romano, Maria Ausilia Catena, Sabrina Cardile, Oliviero Sacco, Donata Girosi, Roberta Olcese, Mariangela Tosca, Giovanni Arturo Rossi, Sergio Salerno, Maria Chiara Terranova, Francesca Santamaria, Angelo Selicorni, Giorgia Mancano, Silvia Maitz, Virginia A. Stallings, Chiara Berlolaso, Carolyn McAnlis, Joan I. Schall, Pasquale Striano, Rita Tanas, Giulia De Iaco, Maria Marsella, Guido Caggese, Paolo Toma, Piero Valentini, Danilo Buonsenso, David Pata, Manuela Ceccarelli, Elvira Verduci, Marta Brambilla, Benedetta Mariani, Carlotta Lassandro, Alice Re Dionigi, Sara Vizzuso, Giuseppe Banderali, Gianvito Panzarino, Claudia Di Paolantonio, Alberto Verrotti, Alberto Villani, Elena Bozzola, Laura Cursi, Annalisa Grandin, Andrzej Krzysztofiak, Laura Lancella, Raffaele Virdis, Patrizia Carletti, Giovanna Weber, Silvana Caiulo, Maria Cristina Vigone

**Affiliations:** 1 Jyväskylä University Psychology Department, Jyväskylä, Finland; 2 Dipartimento di Scienze Mediche Traslazionali, Università degli Studi di Napoli “Federico II”, Napoli, Italy; 3 Pediatric Onco-Hematology, Department of Pediatrics, AOU Città della Salute e della Scienza di Torino, Torino, Italy; 4 Pediatric Endocrinology, Department of Pediatrics, AOU Città della Salute e della Scienza di Torino, Torino, Italy; 5 Transition Unit for Childhood Cancer Survivors, AOU Città della Salute e della Scienza di Torino, Torino, Italy; 6 Regina Margherita Children’s Hospital, Città della Salute e della Scienza, Turin, 10126 Italy; 7 Department of Laboratories, Children’s Hospital Bambino Gesù, Rome, Italy; 8 Pediatric Department, Vimercate Hospital, 20871 Vimercate, MB Italy; 9 Clinica Pediatrica, Dipartimento di Medicina Clinica e Sperimentale, Azienda Ospedaliero-Universitaria di Parma, Università di Parma, Parma, Italy; 10 Study Group of the Italian Society of Pediatrics on the History of Pediatrics, Roma, Italy; 11 Regional Coordinating Centre for Rare Diseases, Academic Medical Center Hospital of Udine, Udine, Italy; 12 Department of Pediatrics and Pediatric Neuropsychiatry, La Sapienza University, Rome, Italy; 13 Pediatric Highly Intensive Care Unit, Department of Pathophysiology and Transplantation, Università degli Studi di Milano, Fondazione IRCCS Ca’ Granda Ospedale Maggiore Policlinico, Milan, Italy; 14 Family paediatrician of Palermo, Palermo, Italy; 15 Primary care teacher School of Specialization in Paediatrics University of Palermo, Palermo, Italy; 16 Terapia Intensiva Neonatale, Azienda Ospedaliera Pugliese-Ciaccio, 88100 Catanzaro, Italy; 17 Departments of Neurology and Pediatrics, Johns Hopkins Hospital, Baltimore, Maryland USA; 18 Pediatric Department University–Hospital, Bambino Gesù Children Hospital, Rome, Italy; 19 Paola Lago, NICU, Women’s and Children’s Health department, Azienda Ospedaliera- University of Padua, Padua, Italy; 20 Elisabetta Garetti, NICU, Women’s and Child’s Health Department, Azienda Ospedaliera-University of Modena, Modena, Italy; 21 Anna Pirelli, NICU MBBM Foundation, San Gerardo Hospital, Monza, Italy; 22 Pediatric Highly Intensive Care Unit, Department of Pathophysiology and Transplantation, Università degli Studi di Milano, Fondazione IRCCS Ca’ Granda Ospedale Maggiore Policlinico, Milan, Italy; 23 Department of Biomedical and Biotechnological Sciences, MMAR Laboratory, University of Catania, Catania, Italy; 24 Pediatric Department, Fondazione MBBM, S. Gerardo Hospital, Monza, Italy; 25 Pediatric Department, S. Martino Hospital, Belluno, Italy; 26 Pediatric Department, S. Anna Hospital, Como, Italy; 27 Department of Pediatrics, “Aldo Moro” University of Bari, Giovanni XXIII Hospital, Bari, 70126 Italy; 28 Chief of SIDS/ ALTE Liguria Center, Pediatric Emergency Department, Giannina Gaslini Children’s Hospital, Genoa, Italy; 29 Department of Pediatrics, Neonatology and Neonatal Pathology, Hospital “G.Fornaroli”, ASST Ovest Milanese, via Al Donatore di Sangue 50, 20013 Magenta, Milan Italy; 30 Department of Science for Health Promotion and Mother and Child Care “G. D’Alessandro” University of Palermo, Palermo, Italy; 31 Unit of Pediatrics, Department of Human Pathology in Adulthood and Childhood “G. Barresi”, University of Messina, Messina, Italy; 32 Pediatric Respiratory and Allergy Units, Giannina Gaslini Hospital and Research Institute, Genoa, Italy; 33 Dipartimento di Biopatologia e biotecnologie mediche, Università di Palermo, Palermo, Italy; 34 Dipartimento di Scienze Mediche Traslazionali, Università Federico II Napoli, Naples, Italy; 35 UOC di Pediatria, ASST Lariana, Como, Italy; 36 UOS Genetica Clinica Pediatrica, Clinica Pediatrica, Fondazione MBBM, Monza, Italy; 37 Gastroenterology, Hepatology and Nutrition, Children’s Hospital of Philadelphia, Philadelphia, PA 19104 USA; 38 Perelman School of Medicine, University of Pennsylvania, Pediatrics, Philadelphia, PA 19104 USA; 39 Department of Pediatrics, Sapienza University, 00161 Rome, Italy; 40 Department of Neurosciences, Rehabilitation, Ophthalmology, Genetics, Maternal and Child Health, University of Genova, Genoa, Italy; 41 UO Pediatria Az Ospedaliero Universitaria, Cona, Ferrara, Italy; 42 Centro per il trattamento dei Disturbi del Comportamento Alimentare, Todi, Perugia, Italy; 43 UOC di Pediatria, Az. Ospedaliera di Rilievo Nazionale “San G.Moscati”, Avellino, Italy; 44 Formazione Professionale, Azienda Ospedaliero Universitaria, Ferrara, Italy; 45 Department of imaging, Bambino Gesù Hospital IRCCS, 00165 Rome, Italy; 46 Department of Health Sciences of the Woman and Child, Pediatrics, “A. Gemelli ” Foundation University Hospital, Rome, Italy; 47 DEA, Pediatric Emergency, Pediatric Hospital “Bambino Gesù”, I.R.C.C.S., Rome, Italy; 48 Department of Specialistic Medicine, Infectious Diseases Specialization School, University of Messina, Messina, Italy; 49 Department of Health Sciences, San Paolo Hospital, University of Milan, Milan, Italy; 50 Department of Pediatrics, University of L’Aquila, L’Aquila, Italy; 51 General Pediatrics and Infectious Disease, Bambino Gesù Children Hospital, Rome, Italy; 52 Board “Immigrants and Health Services” of the Health Commission of The Italian Regions Conference, Rome, Italy; 53 Consultant, GLNBM, Florence, Italy; 54 Coordinator of the National Board. Observatory on Health Inequalities, Health Department, Marche Region, Ancona, Italy; 55 Formerly of Parma University, Parma, Italy; 56 Vita-Salute San Raffaele University, Department of Pediatrics, IRCCS San Raffaele Hospital, Milan, Italy

## A1 Wasted lives, Dreamed lives! Dialogical approach: the value of an inclusive school, prospects and opportunities

### Marco Braghero (marco.braghero@gmail.com)

#### Jyväskylä University Psychology Department, Jyväskylä, Finland

…you have to understand, that no one puts their children in a boat unless the water is safer than the land…Warsan Shire, Home

Child and youth migrations are a particularly dramatic and a daily aspect of the more general problem of contemporary migration flows. Behind and within each of the stories of these children - accompanied and unaccompanied migrant children, as UN calls them in a bureaucratic jargon - school becomes a treasure trove of identity splinters through biographies and fragments of a past that can return visibility to what would be irreparably forgotten otherwise.

School has the opportunity to welcome, support, accompany these children and young new citizens towards the inclusion. School has, also, the opportunity to learn an anthropological view of the presence of migrant children from these life stories, thus activating action-research processes. This action-research will develop new teaching strategies, new approaches based on questions, on an open dialogue, on the paradigms of responsibility, commitment and diversity. A unique opportunity to develop diversity education and citizenship skills, too often mentioned but poorly practiced. Above all, thanks to the sharing and revival of significant life stories and emotionally touching, you can develop emotional intelligence skills, so necessary in an often deregulated age of complexity, which always produces more and more “wasted lives”, above all, thanks to the sharing and reintroduction of significant and emotionally touching life stories.

Through a generous listening of students’ lives, and dialogic practices, school could generate new narrations of migration processes, thus replacing those narrations made up of stereotypical clichés, believes, petty and selfish believes of an overlapping lawlessness, of cruelty and hypocritical welcome. These new narrations tell of possibilities, of mutual discoveries, of processes of successful inclusion, of present-future to build together.

“The dialogical - as Arnkil and Seikkula say - is not a method or a set of techniques but it is an attitude, a way of seeing, which is based on recognizing and respecting the otherness of the other, and on going to meet them.” Applying the integrated dialogic approach to coaching as a lifestyle means to mobilize psychological resources of both people who are directly involved and the whole community and local social network, it means being able to stimulate dialogue.

Stories of wanderings and landings, escapes and refuges, of scared identities and unpredictable cultural metamorphosis, of so much suffering, are intertwined and interdependent to the dreamed and realized stories of successful migrations, fully realized integrations. These new identities are founded and built on a plurality of memberships [1-10].

**References**

1. Shire W. Home. In Lumsden R, Stonborough E (Editors) ‘The Salt Book of Younger Poets’, Noosaville (Australia), Salt, 2011.

2. Bauman Z. Vite di scarto, Bari (Italy), Laterza editore, 2005.

3. MIUR. La via Italiana per la scuola interculturale e l’integrazione degli alunni stranieri-ottobre 2007. Accessed at http://hubmiur.pubblica.istruzione.it/web/istruzione/intercultura-normativa (04/11/2016).

4. Arnkil ET, Seikkula J. Metodi dialogici nel lavoro di rete. Gardolo (Trento, Italia), Erickson, 2013.

5. MIUR. Linee guida per l’accoglienza e l’integrazione degli alunni stranieri- febbraio 2014. Accessed at http://hubmiur.pubblica.istruzione.it/web/ministero/focus190214 (04/11/2016).

6. Di Nuzzo A. Fuori di casa. Migrazioni di minori non accompagnati. Roma (Italia), Carocci editore, 2014.

7. Sistema di Protezione per Richiedenti Asilo e Rifugiati, Atlante SPRAR 2015. Accessed http://www.sprar.it/index.php?option=com_k2&view=item&id=45:rapporti-annuali-e-compendi-statistici-dello-sprar&Itemid=553 (04/11/2016).

8. Santagati M, Ongini V. Alunni con cittadinanza non italiana. La scuola multiculturale nei contesti locali, Rapporto nazionale A.s. 2014/2015. Milano, Fondazione ISMU, 2016.

9. UNICEF. “Uprooted: the growing crisis for refugee and migrants children”, New York (US), UNICEF, 2016.

10. Fondazione ISMU. Sbarchi 2016: aumentano i minori non accompagnati, 21 settembre 2016. Accessed at http://www.ismu.org/minori-stranieri-non-accompagnati/ (04/11/2016).

## A2 Management of acute diarrhoea

### Annamaria Staiano (staiano@unina.it)

#### Dipartimento di Scienze Mediche Traslazionali, Università degli Studi di Napoli “Federico II”, Napoli, Italy

Diarrhoea in children still has a major impact on health-related social costs, affecting approximately 2 billion children younger than 5 years every year, and determining 2 million deaths, mostly in Africa and Asia. [1]

According to WHO diarrhea consists in ≥*3 passages of softened or liquid stools within 24 hours*. Acute forms (duration 0-7 days) are traditionally defined in distinction with protracted (7-14 days) and chronic (>14 days) diarrhoea.

ESPGHAN guidelines [2] state that acute gastroenteritis (AGE) does not generally require a specific diagnostic work-up. Microbiologic investigations should be limited to subjects with chronic diseases, in severely compromised conditions or with long-lasting symptoms that could be potentially eligible for specific treatments. The assessment of the degree of dehydration still remains the cornerstone of the management. Newborns and children aged less than 2 months, subjects with severe conditions, persistent vomit or massive diarrhoea (>8 episodes/day) should be always clinically assessed. Hospital admission should be considered in cases of shock, severe dehydration, neurological abnormalities, intractable or bilious vomit, oral rehydration failure, when a surgical condition is suspected or when parental management at home does not represent a safe option.

The distinction between bacterial and non-bacterial etiologies is not relevant to the treatment: the basic therapy is oral rehydration [3]. Sometimes oral rehydration is not sufficient and i.v. fluids may be required (shock, altered level of consciousness, severe acidosis, failure of oral/enteral rehydration, persistent vomit, abdominal distension or ileus).

In children, AGE treatment may include the use of several drugs (antiemetics, probiotics, anti-secretory drugs, gelatin tannate).

Antiemetics decrease need for hospitalization, but may entail electrocardiographic alterations (i.e. prolonged QT interval) [2].

A recent Cochrane review [4] concluded that probiotics may have a role in decreasing duration of diarrhoea of approximately one day, in reducing stool frequency during the second day and the risk of diarrhoea lasting longer than 4 days. Even though some “strong recommendations” support the use of some specific strains (*Lactobacillus GG* and *Saccharomyces boulardii*), quality of evidence in favour of probiotics is generally low [4, 5].

Data regarding diosmectite and racecadotril should be carefully interpreted, as most of the available studies present major drawbacks [6, 7].

Finally, some evidence supports the use of gelatin tannate, a “mucosal regenerator” that creates a layer adhering to the intestinal wall which can protect against the penetration of aggressive bacteria [8, 9].

**References**

1. Black R, Allen LH, Bhutta ZA, et al. Maternal and child undernutrition: global and regional exposures and health consequences. Lancet 2008; 371:243-260.

2. Guarino A, Ashkenazi S, Gendrel D, et al. European Society for Pediatric Gastroenterology, Hepatology, and Nutrition/European Society for Pediatric Infectious Diseases evidence-based guidelines for the management of acute gastroenteritis in children in Europe: update 2014. J Ped Gastroenterol Nutr. 2014; 59: 132–152.

3. Fontaine O, Garner P, Bhan MK. Oral rehydration therapy: the simple solution for saving lives. BMJ. 2007; 334 Suppl 1: s14.

4. Goldenberg JZ, Lytvyn L, Steurich J, et al. Probiotics for the prevention of pediatric antibiotic-associated diarrhea. Cochrane Database Syst Rev. 2015;12 CD004827: 1-93.

5. Szajewska H, Skórka A, Ruszczyński M. Meta-analysis: Lactobacillus GG for treating acute gastroenteritis in children--updated analysis of randomised controlled trials. Aliment Pharmacol Ther. 2013;38:467-476.

6. Szajewska H, Dziechciarz P, Mrukowicz J. Meta-analysis: Smectite in the treatment of acute infectious diarrhoea in children. Aliment Pharmacol Ther. 2006;23:217-227.

7. Gordon M, Akobeng A. Racecadotril for acute diarrhoea in children: systematic review and meta-analyses. Arch Dis Child 2016;101:234-240.

8. Frasca G, Cardile V, Puglia C, et al. Gelatin tannate reduces the proinflammatory effects of lipopolysaccharide in human intestinal epithelial cells. Clin Exp Gastroenterol. 2012;5:61-67.

9. Lopetuso LR, Scaldaferri F, Bruno G, et al. The therapeutic management of gut barrier leaking: the emerging role for mucosal barrier protectors. Eur Rev Med Pharmacol Sci. 2015;19:1068-1076.

## A3 Growth deficit in childhood cancer survivors

### Eleonora Biasin^1^, Patrizia Matarazzo^2^, Silvia Einaudi^2^, Rosaria Manicone^1^, Francesco Felicetti^3^, Enrico Brignardello^3^, Franca Fagioli^1^

#### ^1^Pediatric Onco-Hematology, Department of Pediatrics, AOU Città della Salute e della Scienza di Torino, Torino, Italy; ^2^Pediatric Endocrinology, Department of Pediatrics, AOU Città della Salute e della Scienza di Torino, Torino, Italy; ^3^Transition Unit for Childhood Cancer Survivors, AOU Città della Salute e della Scienza di Torino, Torino, Italy

##### **Correspondence:** Eleonora Biasin (eleonora.biasin@unito.it)

Survival after childhood cancer has substantially improved during the past decades, and it is now up to 80% at 5 years, considering all diseases. The number of long-term survivors rises every year; these patients show an increase of late morbidity and mortality with negative impact on the quality of life. It is well known that two -thirds of longterm survivors will have at least one chronic illness related to prior treatment and in one-third of the cases, the pathological alteration will be so serious to lead to potentially disabling or life-threatening diseases [1].

Both chemotherapy and/or radiotherapy (RT) in the previous treatment can be responsible of longterm diseases. In particular, central nervous system RT can cause a hypothalamic-pituitary axis damage. The secretion of growth hormone (GH) is frequently affected by the negative effects of RT [2]. In fact, delay and growth deficit have been evidenced starting from a RT dose of 4 Gy that can be responsible of vascular and/or neuronal damage. Furthermore, there is a positive correlation between total radiation dose and time since treatment and pituitary hormones deficiency [3].

Considering the treatment used in the last and more recent years, the patients that need more growth surveillance are the ones affected by acute lymphoblastic leukemia (when central nervous system RT is administered), brain tumor, nasopharyngeal carcinoma, hypothalamic-pituitary tumor and patients undergoing hematopoietic stem cell transplant when total body irradiation is administered as conditioning regimen.

All these patients should undergo a follow up. When the growth velocity is persistently below 10° centile the patient should undergo tests to evaluate GH secretion. If there are two pathologic tests the use of GH replacement is recommended at physiological dose and targeting somatomedin level, starting at least 2 years after the end of oncologic treatment.

The increase of second tumor incidence in childhood cancer survivors after GH treatment is nowadays under discussion while an increase in incidence of relapse has not been confirmed. These data should be evaluated considering that an increase of risk of developing second tumor has also been observed after RT, and this could affect the data obtained in some studies of GH replacement [4-7].

**References**

1. Oeffinger KC, Mertens AC, Sklar CA, Kawashima T, Hudson MM, Meadows AT, et al. Childhood Cancer Survivor Study. Chronic health conditions in adult survivors of childhood cancer. N Engl J Med. 2006; 355: 1572-1582.

2. Darzy KH, Shalet SM. Hypopituitarism following Radiotherapy Revisited. Endocr Dev. 2009; 15: 1-24.

3. Follin C, Erfurth EM. Long-Term Effect of Cranial Radiotherapy on Pituitary-Hypothalamus Area in Childhood Acute Lymphoblastic Leukemia Survivors. Curr Treat Options Oncol. 2016; 17: 50.

4. Brignardello E, Felicetti F, Castiglione A, Fortunati N, Matarazzo P, Biasin E, et al. GH replacement therapy and second neoplasms in adult survivors of childhood cancer: a retrospective study from a single institution. J Endocrinol Invest. 2015; 38: 171-176.

5. Woodmansee WW, Zimmermann AG, Child CJ, Rong Q, Erfurth EM, Beck-Peccoz P, et al. Incidence of second neoplasm in childhood cancer survivors treated with GH: an analysis of GeNeSIS and HypoCCS. Eur J Endocrinol. 2013;168:565-573.

6. Raman S, Grimberg A, Waguespack SG, Miller BS, Sklar CA, Meacham LR, Patterson BC. Risk of Neoplasia in Pediatric Patients Receiving Growth Hormone Therapy--A Report From the Pediatric Endocrine Society Drug and Therapeutics Committee. J Clin Endocrinol Metab. 2015; 100: 2192-2203.

7. Patterson BC, Chen Y, Sklar CA, Neglia J, Yasui Y, Mertens A, et al. Growth hormone exposure as a risk factor for the development of subsequent neoplasms of the central nervous system: a report from the childhood cancer survivor study. J Clin Endocrinol Metab. 2014; 99: 2030-2037.

## A4 Transition: a multidisciplinary glance

### Elisabetta Bignamini, Elena Nave

#### Regina Margherita Children’s Hospital, Città della Salute e della Scienza, Turin, 10126, Italy

##### **Correspondence:** Elisabetta Bignamini (ebignamini@cittadellasalute.to.it)

During the last decade, the role of transitional care in subjects affected by chronic complex diseases acquired great relevance and national and international journals published articles about this topic.

The technological and therapeutic evolution, in fact, allowed children who previously died during childhood, to reach adulthood, with the apparition of a different and “new” adult patient, young, but chronically ill, with a long history of illness.

Actually it is not clear if the interest on this theme was born from specific medical reasons, like the difference between competences and knowledge of pediatricians and physicians who take care of adults, or from the health care system organization or, besides, for answering to a social cultural question, including the needs of patients and families.

In order to answer these questions, it is important to focus, in a multidisciplinary glance, on some basic concept like “child”, “transition” and “adult”. Society itself acquired a structure that took into account the sharp demarcation between children and adults, enhancing activities specifically devoted to the two.

The transition or “passage”, from an anthropological point of view, has been studied by the ethnologist Van Gennep [1] at the beginning of XX century who reported a characteristic tripartite dimension, with an important intrinsic role in shaping the participants:

- Separation (pre-liminal phase): the part of the ritual in which the “participant” is separated from the social group which he/she belongs;
- Transition (liminal phase): the participant is placed in a social “limbo” in which he/she is outside the group he belonged to, but also outside the group he/she is going to place in;
- Reinstatement (post-liminal phase): the participant is in the new group

“Transition”, in bio-medicine, has been defined like “the purposeful, planned movement of adolescents and young adults with chronic physical and medical conditions from child centered to adult – oriented health care systems” [2]

It is clear that we must follow “a ritual practice”, that on one side, needs standardized itineraries, on the other must be costumed to that particular patient in his/her socio-cultural context. Some chronic respiratory diseases, for example Cystic fibrosis, have evolved models and standards of care for transition.

There are also ethical implications in an unsatisfactory transition: young people and their family could remain entrapped in an undefined dimension that influences their perception of illness and wellbeing.

**Acknowledgements**

The Authors thank Ilaria Lesmo (anthropologist) for her previous work on this topic.

**References**

1. Van Gennep A. I riti di passaggio. Torino: Bollati Boringhieri, 2002 [ed. orig. Les rites de passage. Paris: É. Nourry, 1909].

2. Blum RW, Garrell D, Hodgman C. Transition from child-centered to adult-oriented care: systems for adolescents with chronic health conditions. J Adolesc Health. 1993; 14: 570-576.

## A5 A cooperation model of the Children Hospital Bambino Gesù (OPBG) in Tanzania

### F. Callea, C.Concato, E. Fiscarelli, S.Garrone, M. Rossi de Gasperis

#### Department of Laboratories, Children’s Hospital Bambino Gesù, Rome, Italy

##### **Correspondence:** F. Callea (francesco.callea@opbg.net)

In the late 90s epidemic/endemic AIDS had caused million deaths, mostly young adults, and an impressive number of children orphans of both parents. No prevention or educational campaign, no drugs were available in Tanzania at that time.

In 2002 two Italian missionaries founded the Village of Hope to welcome and to accompany diseased bi-orphan children to pass away.

One year later, the missionaries had a fortunate access to antiretroviral drugs and administered them to all seropositive children. Since then no single child died. Later on the antiretroviral treatment was extended to mono-orphans and parent as out-patients and subsequently to pregnant women according to WHO recommendation.

In this scenario OPBG has found a rich soil for an innovative model of cooperation: establish a laboratory for HIV test, CD4 count, viral load and drug resistance, by providing instruments, reagents and personnel who was moving upon rotation from the Central Lab in Rome to the Village of Hope. By doing that, OPBG realized an Institutional Voluntary Service under the motto “to badge at Southern Equator”, a P.O.C.T. and Test and Treat Task (T.T.T.) system.

The results (2005-2013) are remarkable: 146/170 children alive, 67 of which treatment-free, 51 with less than 50 viral copies, 130/198 HIV-free neonates at 18 mo follow-up.

Children and adolescents by the time were developing all pediatric diseases requiring medical and surgical specialties (infectivologists, dermatologists, surgeons etc.) mostly provided by OPBG. Presently the Village of Hope behaves as a children’s hospital (Policlinics) for in-patient and out-patients, the latter being now more than 2000. So far this is the first example of hospital departments driven by laboratory services.

The results obtained in terms of survival and good health have generated the need for continuity of cure and care and the intuition of providing education. Primary and secondary schools have been activated within the Village and adolescents have remained inside the Village thus preventing them to returning to be “niños de ruas”.

For the whole above the OPBG cooperation model is designated “Laboratory for Human Promotion”.

Finally the OPBG Laboratory activity has led to scientific publications in International Journals [1,2], thus proving that humanitarian interventions can generate also research activities.

**References**

1. Rossi de Gasperis M. et al “Quantitative recovery of proviral HIV-1 DNA from leukocytes by the Dried Buffy Coat Spot method for real-time PCR determination”.J Virol Methods. 2010;170:121-7.

2. Meini G. et al “Frequent detection of antiretroviral drug resistance in HIV-1-infected orphaned children followed at a donor-funded rural pediatric clinic in Dodoma, Tanzania”, AIDS Res Hum Retroviruses. 2015 ;31:448-51.

## A6 Participants networks’ observations

### Patrizia Calzi, Grazia Marinelli, Roberto Besana

#### Pediatric Department, Vimercate Hospital, 20871 Vimercate (MB), Italy

##### **Correspondence:** Patrizia Calzi (patrizia.calzi@asst-vimercate.it)

Pediatric network proved to be a good instrument to monitor the clinic management of baby with bronchiolitis, showing strengths and weaknesses to which improvement measures should be addressed [1, 2]. The major critical issue, despite the capillarity of information, has been the poor adhesion of the hospitals. One of the main reasons is the lack of staff dedicated to the regular collection of data: to be effective, a network needs a strongly integrated organization. Some important lessons can be discerned from our Network (Table 1).

**Conclusions**

The project, in order to be helpful for clinical and organizational choices, needs to obtain a larger participation. For greater effectiveness, a national coordination is required, as much as the support given by scientific societies with approved guidelines.

**Acknowledgements**

We thank all the members participating in the project (Table 2).

**References**

1. Wasserman R. Pediatric Clinical Research. Networks: optimizing effectiveness through cooperation. Accessed at: http://grantome.com/grant/NIH/R13-EY019972-01A1 (04/11/2016)

2. Ralston SL, Garber MD, Rice-Conboy E et al. A multicenter collaborative to reduce unnecessary care in inpatient bronchiolitis. Pediatrics. 2016; 137:e20150851

**Table 1 (abstract A6).** Learning from our experience

Need of a wide number of pediatric departement	
Integrated organization	
Better communication platform for partner to improve collaboration	
Providers have to deliver the best care	
Periodic information and feedback is essential to maintain high the level of interest

**Table 2 (abstract A6).** Centers participating in the project

Pediatric Departement - Hospital	Country
Anna Rizzoli – Ischia (Na)	Campania
Vizzolo Predabissi - Melegnano (MI)	Lombardia
ICP- Milano	Lombardia
Ponte San Pietro (BG)	Lombardia
Sant’Anna - Como	Lombardia
Fornaroli - Magenta (MI)	Lombardia
Vimercate (MB)	Lombardia
San Bortolo - Vicenza	Veneto
Saronno (VA)	Lombardia
Legnano (MI)	Lombardia

## A7 What’s behind editorial office working

### Carlo Caffarelli, Antonio Di Peri, Irene Lapetina

#### Clinica Pediatrica, Dipartimento di Medicina Clinica e Sperimentale, Azienda Ospedaliero-Universitaria di Parma, Università di Parma, Parma, Italy

##### **Correspondence:** Carlo Caffarelli (carlo.caffarelli@unipr.it)

A few key figures are responsible for the quality of a scientific article collaborating closely in the editorial process: authors, publisher, reviewers and technical staff. When a journal receives a paper for potential publication, coworkers of the editorial office check that the authors have diligently followed the instructions on how to style the text and where needed they declare approval of the Ethical Committee, as well as have received the permission to publish material already appeared in other articles. If there is the suspicious of plagiarism, the editorial team uses an IT system to verify it. Subsequently, the editor ensures the content and the process of the publication itself. He is responsible for the quality of the paper as well as for the soundness of the information. For this purpose, he collaborates with reviewers, usually two. Reviewers check the quality of the proposed article, and are precious and anonymous contributors to the authors' work. Choosing the right reviewers is a step the publisher is responsible for. When doing so, he takes into consideration any previous relevant experience with the topic to be assessed as well as their availability to deliver an accurate and competent review on time. It’s also important to have a broad network of reviewers. It should not be limited by the editor’s usual collaborators but it should also include expert reviewers whose work can increase the audience of the journal. The reviewers should always maintain a collegial attitude, "educational" and "constructive". Finally it may be necessary to look for technical reviewers to revise statistical or bioinformatics analysis. After evaluating comments and opinions of the two reviewers the Editor may: 1) accept the job; 2) send it to the authors requesting to amend the corrections by the two reviewers; 3) in the case of disagreement ask for a second opinion or make a decision himself. Subsequently, the editor will review together with the reviewers the second amends made by the authors. If the authors have responded adequately the work is accepted. If not, the authors have a last chance to review it before final rejection. Once the article has been accepted, the correction team will help reviewing the spelling, the vocabulary and the graphic design. In conclusion, for a correct and comprehensive publication it is necessary a close cooperation and collaboration among authors, editor and reviewers in order to ensure the quality of the article.

## A8 Giuseppe Mya, Master of Pediatrics in Florence

### Patrizia Cincinnati (patnati@mclink.net)

#### Study Group of the Italian Society of Pediatrics on the History of Pediatrics, Roma, Italy

**Background**

Almost 160 years after the birth of Giuseppe Mya (1857-1911), the founder of the Florentine pediatric School, we have reviewed his research in order to evaluate its impact on child health care and pediatric knowledge during that period.

**Materials and methods**

We examined five prestigious national medical journals of the time, from 1891- when Giuseppe Mya was called at the Institute of Higher Studies in Florence - until his death: *Lo Sperimentale*, *Il Policlinico*, *La Pediatria*, *Rivista di Clinica pediatrica* and *Rivista di Patologia nervosa e mentale*. We also reviewed the Proceedings of the first six Pediatric Italian Congress. From these sources we obtained information about the author's major scientific contributions as well as his approach to the problems of the emerging pediatric community in Italy.

**Results**

With regard to diphtheria - a troubling childhood disease at that time - Mya was one of the first European supporters of the antidiphtherial serum (1894) and opened new challenging pathogenetic perspectives about bronchopneumonia (1895), paralysis (1899) and non-obstructive emphysema (1908) associated to the illness.

Also the term of Congenital Megacolon and its nosographic framing date back to Mya (1894), as well as the first report about a family case of congenital Hydrocephalus (1896). The description of the abnormalities in cerebro-spinal fluid of the children suffering from tuberculous meningitis too dates back to Giuseppe Mya (1897).

Mya’s perspectives were constantly anchored to pathological and experimental findings. The Master encouraged his collaborators (Carlo Comba, Dante Pacchioni, Carlo Francioni) to perform further research according to this methodology and his scientific rigor led them to the highest levels of quality. Interestingly, Mya criticized the separation between clinical and laboratory activities (1905), thus anticipating the successful strategy "From the bedside to the bench and back".

The Master's sensitivity for the disadvantaged social conditions favoring infant diseases is also evident. From this point of view the investigation performed with his staff on the state of childhood in Florence is enlightening (1909).

Founding member and later the President of the Italian Society of Pediatrics, co-editor of *Monatsschrift für Kinderheilkunde* and co-founder of *Rivista di Clinica Pediatrica*, his interventions to the Congress show a constant concern for the promotion of pediatric culture in the country through a qualified mandatory academic teaching favored by institutional and economic measures (1901, 1905, 1907).

**Conclusions**

Giuseppe Mya was a pioneer of the modern Italian pediatrics being a reference for all contemporaries interested in infancy diseases.

## A9 Child migration phenomenon in Italy

### Rosalia Maria Da Riol (rosidariol@gmail.com)

#### Regional Coordinating Centre for Rare Diseases, Academic Medical Center Hospital of Udine, Udine, Italy

In recent years, in the context of migratory flows affecting Europe and Italy in particular, the juvenile component is increasingly numerically important, heterogeneous and constantly evolving. Migrant minors (MM) in our country, as of 31.12.2014, were 1,085,274 (21.6% of the foreign resident population); of these, in 2014, 75,067 were born in Italy from two foreign parents (14.9% of total births) [1]. In the latest decades, these "foreign newborns" have helped reduce the negative demographic trend of the Italian population which is still aging dramatically.

In Italian schools, in 2014/2015, 9.2% of pupils were not Italian citizens (814,187), even if 55.3% were born in Italy [2]. In recent years, the increase of adolescents arriving in Italy as a result of family reunification and the increase of born to non-Italians have led to an ever growing importance of foreign students in the second cycle of education, not only in primary and lower secondary levels.

To these MMs other typologies are added, such as children adopted abroad, Roma/Sinti minors living in “nomad camps”, the children of refugees and Unaccompanied Migrant Minors (UMMs). The latter, coming from countries affected by war and persecution, have represented over the last three years a constantly evolving dramatic phenomenon (11,921 UMMs as of 31.12.2015, 13.1% more than in 2014; 12,241 UMMs as of 30.06.2016) [3].

Each type of MM is characterized by a specific migration history (country of origin and family situation, trip type, reception/permanence conditions) and by the consequent exposure to socioeconomic and environmental risk factors that affect the health framework of the child and its special care needs [4]. The situation of vulnerability and fragility of these MMs can be further aggravated by irregularities in the legal status of their parents, which affects their access to dedicated health services and, in particular, the ongoing support of Primary Care Paediatricians (PCPs). Although the State-Regions Agreement n.255/2012 [5] has introduced obligatory enrollment in the NHS and PCPs for undocumented MMs, in many Italian regions this is not done denying them the right to health as a state of complete physical, mental, social wellbeing.

In this context, the role of the paediatrician, regardless of the type of MM, cannot be just one of treatment but also of advocacy and supervision in the design and implementation of public health policies that protect the right to health of these children with a view to fairness and inclusion.

**References**

1. Dossier Statistico Immigrazione UNAR IDOS 2015

2. Rapporto nazionale MIUR ISMU 2014/2015 Accessed at: http://www.istruzione.it/allegati/2016/Rapporto-Miur-Ismu-2014_15.pdf (04/11/2016)

3. Ministero del Lavoro e delle Politiche Sociali. Report Nazionale Minori stranieri non accompagnati in Italia. Report di monitoraggio dei dati censiti al 30 giugno 2016. Accessed at http://www.lavoro.gov.it/temi-e-priorita/immigrazione/focus-on/minori-stranieri/Pagine/default.aspx (04/11/2016)

4. Nuove indicazioni del GLNBM-SIP per l’accoglienza sanitaria al minore migrante. 2013. Accessed at http://www.glnbi.org (04/11/2016)

5. Accordo Stato-Regioni n. 255 del 12 dicembre 2012. Indicazioni per la corretta applicazione della normativa per l’assistenza sanitaria alla popolazione straniera da parte delle Regioni e Province autonome. Accessed at: http://www.statoregioni.it/Lista_Documenti.asp?Pag=2&DATA=20/12/2012&CONF=CSR (04/11/2016)

## A10 Extreme therapeutic choices and therapeutic obstinacy: extremely low birth weight infants and late-term abortion survivors

### Mario De Curtis, Lucia Dito, Chiara Protano

#### Department of Pediatrics and Pediatric Neuropsychiatry, La Sapienza University, Rome, Italy

##### **Correspondence:** Mario De Curtis (mario.decurtis@uniroma1.it)

**Background**

The number and survival rates of extremely low birth weight (ELBW) infants born with a gestational age (GA) <26 weeks have increased in the last few years. Given their severe immaturity, these infants are inevitably exposed to critical conditions leading to death or to severe disabilities, and this is posing major bioethical concerns in many countries.

An additional problem emerging recently in Italy is the medical care of late-term abortion survivors. When abortion became legal in Italy, in 1978, the survival threshold for premature infants was 24-25 weeks GA. Today, the therapeutic progress has progressively reduced the threshold to 22 GA weeks, leading to new, quite challenging bioethical problems. In Italy, the termination of pregnancy beyond 90 days GA is legal only if the mother's life is endangered or in case of severe fetal abnormalities compromising the mother’s physical or mental health; it is nevertheless illegal if there are chances of autonomous fetal life. Gestational age is nowhere mentioned.

**Materials and methods**

To define the extent of the problem, we searched the Italian Vermont Oxford Network (VON) to calculate the survival rates of 163 infants born at 22 and 23 weeks GA, cared for in 32 Italian NICUs in 2013. We also analyzed the number of abortions and of infants born from late-term abortions (>21 weeks) in 2015 in the Lazio Region.

**Results**

Based on VON data, the survival of the 30 infants born at 22 weeks GA was 23% and of the 130 born at 23 weeks GA, 32%. In 2015, in Lazio, 9617 abortions were performed (10% of all abortions in Italy), of which 55 at 22 weeks GA, 19 at 23 weeks and 3 subsequently.

**Conclusions**

Since pre-term infants may survive even at 22-23 weeks GA, our question is whether it is ethically justifiable to provide care for an infant surviving an abortion carried out at 22-23 weeks GA, given the very high risk of severe malformations and disabilities, whose mother has decided to terminate her pregnancy. Some find it cruel, others believe that a viable fetus should always be reanimated. A neonatologist cannot be left alone in deciding what to do, only to be charged with either medical neglect or, vice versa, therapeutic obstinacy.

## A11 Influenza and surroundings

### Susanna Esposito (susanna.esposito@unimi.it)

#### Pediatric Highly Intensive Care Unit, Department of Pathophysiology and Transplantation, Università degli Studi di Milano, Fondazione IRCCS Ca’ Granda Ospedale Maggiore Policlinico, Milan, Italy

Influenza is a common disease. Up to 30% of children, with the highest prevalence among the youngest, are infected by influenza viruses every winter season. Most of the disease cases are mild and spontaneously resolve; however, influenza in children can be severe enough to lead to hospitalization and death. In the last four influenza seasons in the USA, a total of 515 influenza-associated paediatric deaths have occurred. However, it is highly likely that the true impact of influenza infection in paediatrics is significantly higher than what is reported in epidemiological studies and official statistics. This is because a great number of patients who are hospitalized and die from severe respiratory problems because of influenza infection are not tested for influenza viruses. Moreover, influenza infection is often not reported as the contributory cause of hospitalization or death when the main signs and symptoms of disease are strictly related to the worsening of an existing chronic underlying illness. For many years, it was thought that severe influenza cases were only common in children at high risk of influenza-related complications because of a chronic, severe, underlying disease. Consequently, the prevention of influenza through the use of available influenza vaccines was only recommended to these individuals. However, in recent years, several studies have shown that severe cases can occur in otherwise healthy children. This explains why several countries currently recommend universal influenza vaccinations in children. Because the highest risk of hospitalization and death in otherwise healthy children occurs in the first years of life, a number of countries limit vaccination recommendations to infants, toddlers and preschool children. In other cases, such as in the USA, influenza vaccine administration is recommended in all paediatric populations from 6 months to 17 years and also in adults of any age. For years, protection against influenza has been pursued by administering the trivalent inactivated vaccine given intramuscularly. More recently, quadrivalent inactivated and live attenuated vaccines were prepared and licensed. Quadrivalent vaccines appear extremely attractive for the pediatric population due to the relevance of influenza B in children. However, because these preparations cannot be used in younger infants, different immunization methods to protect these subjects have been pursued. Maternal immunization is one of these methods, although presently it is not adequately used. Knowledge on influenza vaccination should be increased in order to adequately protect children against a common disease that may cause severe complications.

## A12 Area pediatrica round table: deeds and misdeeds. Extreme food choices and nutritional fads

### Dante Ferrara^1,2^ (ferraradnt@libero.it)

#### ^1^Family paediatrician of Palermo, Palermo, Italy; ^2^Primary care teacher School of Specialization in Paediatrics University of Palermo, Palermo, Italy

Recent reports have pointed out cases of hospitalised paediatric patients, in serious clinical conditions, because of an improper food regime, linked to the spread of diets different to the omnivorous one. The dimensions of the problem and the related ethical implications impose it to scientific attention.

**Fig. 1 (abstract A12).**
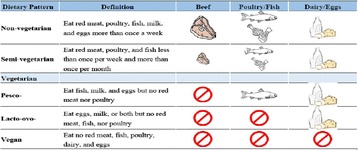
Classification of food styles [1, 3]

On a terminological point of view, these diets are distinguished in: vegan diet, which is exclusively based on the consumption of vegetable food (Fig. 1) [1, 2]; lacto-ovo-vegetarian (LOV) diet, which is based on the consumption of vegetable food and indirect animal food (eggs, cow’s milk and by-products, honey) (Fig. 1) [1, 3]; raw food diet, which is based on the consumption of raw food; macrobiotic diet, suggested by the Japanese doctor Nvioti Sakurawa, which is part of an Eastern-inspired lifestyle based on a balance between Yin (associated with acidic foods) and Yang (associated with alkaline foods).

At a demographic level, Eurispes data point out that about the 8% of the Italian population follows an alternative diet, the 7.1% a vegetarian one and the 0.9% a vegan one [4, 5].

In Italian families vegetarian or vegan food choices depend in the 31% of the cases on ethical reasons (animals’ defence), in the 46.7% of the cases on reasons linked to health safeguard; in the remaining cases, it depends on religious, philosophical, economic and environmental reasons [3].

About the second data, several scientific societies [6-9] have actually recommended the Mediterranean diet and a reduced consumption of red meat, to the advantage of cereals, legumes and vegetables. On this basis, however, poorly conscious parents have extended extreme food choices to their young children. In these cases, paediatricians’ support is indispensable.

Vegan or vegetarian diets in a fast growing organism such as the child one involve biochemical-preclinical or frankly clinical deficiency conditions [10-13], if not properly supported by supplements of proteins (10% -15% more than the recommended consumption), of vitamin B12 (from 5 to 50 mcg/die depending on the age), of iron (1.8 times more than non-vegetarians), of zinc (50% more than the recommended dose), of iodine (3 gr/die of iodized salt after the first year or of possible supplements later), of calcium and ω-3 e ω-6 fatty acids (appropriate dietary adjustments).

It is therefore dangerous for paediatricians to refuse the alternative diets proposed by parents, thus leaving them freedom in the young patient nutrition field; on the contrary, they must be able to provide guidance on the correct supplements in order to ensure the compatibility of these diets with a balanced psycho-physical growing of the child.

**References**

1) Accessed at: http://webcache.googleusercontent.com/search?q=cache:HmgYBlrlah4J:www.leurispes.it/vegetariani-vegani-alimentazione-futuro/+&cd=1&hl=it&ct=clnk&gl=it.

2) EURISPES. Accessed at: http://www.greenstyle.it/ (04/11/2016) htpp://www.greenstyle.it/alimentazione-un-italiano-su-14-vegetariano-o-vegano-71093.htm (04/11/2016)

3) FAO, Livestock's long shadow.

4) Le LT, Sabaté J. Beyond Meatless , the Health Effects of Vegan Diets: Findings from the Adventis Cohorts. *Nutrients* 2014, 6, 2131-2147.

5) De Gana L. Vegetariani e vegani- attualità e prospettive scientifiche, Relazione EXPO Milano, 28 giugno 2015. 6) Ferrari ML, Berveglieri M. Alimentazione vegetariana in pediatria. Medico e Bambino. 2015; 34:165-169.

7) Craig WJ, Mangels AR, American Dietetic Association. Position of the Academy of Nutrition and Dietetics: Vegetarian diets. J Am Diet Assoc. 2009;109:1266-82.

8) USDA (United States Department of Agriculture). Dietary guidelines for americans. 2010: chapter 5: building healthy eating patterns. Accessed at http://www.fns.usda.gov/sites/default/files/Chapter5.pdf (04/11/2016).

9) American Academy of Pediatrics. Committee on Nutrition.Nutritional Aspects of Vegetarianism Health Foods, and Fad Diets. Pediatrics. 1977.59,460

10) Berveglieri M. La dieta vegan in età pediatrica. Alimentazione salutare per i bambini e le famiglie secondo l'evidenza scientifica. Vegan Festival 7/8 maggio 2016 Ferrara.

11) Pinelli L. Bambini e alimentazione vegetariana TERRAE’ Ecologia della nutrizione e i 5 colori della salute, Pordenone 12 maggio 2012.

12) Gallo P, Lambertini A, Landini C, et al. Quando una vitamina fa la differenza. Medico e Bambino. 2016;35:231-236 .

13) Gastaldi R, Panicucci C, Poggi E, et al. La carenza di iodio in età evolutiva Medico e Bambino. 2015;34:39-43.

## A13 Forensic aspects and reporting ultrasound criteria

### Rossella Galiano, Pasquale Novellino

#### Terapia Intensiva Neonatale, Azienda Ospedaliera Pugliese-Ciaccio, 88100 Catanzaro, Italy

##### **Correspondence:** Rossella Galiano (ferraradnt@libero.it)

Exposure to ultrasound (US), at normal diagnostic level, has no adverse biological effects. Therefore the sonogram, unlike other imaging techniques, is not regulated by specific laws and its use is not limited to imaging specialist, but is allowed for all the physicians. The specific features (non-invasive, painless, repeatable) associated to actual availability of equipment with excellent performance, always smaller (handheld), inexpensive, have led to the great success of this methodology and its rapid spread. The increasing demand for pediatric US has been faster than ability of the institutions to train and accredit medical sonographers and to plan protocols, guidelines or recommendations .The inevitable consequence has been a great heterogeneity of competence, skills and experience of practitioners using ultrasound, which is, by definition, a user-dependent technology. In a cultural environment that emphasizes the performance of diagnostic tools and undervalues their limitations, the inevitable effect is a medico-legal disputes increasing. Since ultrasound is a noninvasive technique, disputes resulting from damage provoked during ultrasound examinations (mostly eco guided interventional maneuvers) are rare; almost always disputes stem from a wrong or missed diagnosis when this was possible (" not prevent an event that has a legal obligation to prevent is equivalent to causing it"). During the trial the skill, prudence and diligence of sonographers will be judged according to iconographic documentation and the report (description of what was seen and diagnostic interpretation). Iconographic documentation and report are an integral part of the performance. Both have important legal significance, because while the diagnostic error may fall within the risk inherent in any medical activity, and it not always is a guilt, inadequate iconographic documentation or incomplete report are always marker of negligent professional performance. Different is the case of “Bedside” or “Office” or “Point of care” ultrasound, examination carried out and interpreted directly by clinician, as integration of objective examination. This kind of US respond to a specific diagnostic question or facilitates interventional maneuvers , without pretending to replace a comprehensive imaging carried out by imaging specialists. Currently in Italy there are no laws regulating the use of bedside US. To ensure the quality of pediatric ultrasound is necessary dedicated training, accreditation programs, guidelines and recommendations, and specific rules to distinguish the responsibilities of those who perform ultrasound in outpatient dedicated services and those who practice the bedside US.

## A14 Chetogenic diet: evidence of efficacy

### Eric Heath Kossoff (ekossoff@jhmi.edu)

#### Departments of Neurology and Pediatrics, Johns Hopkins Hospital, Baltimore, Maryland, USA

The ketogenic diet has been in continuous use since 1921 and has in the past two decades grown dramatically in popularity. It is used primarily for children with very severe epilepsy not responsive to at least two standard anticonvulsant medications. However, recent years have seen the emergence of use earlier in the course of epilepsy, for adolescents and even adults, and also for neurologic conditions other than epilepsy (including cancer, Alzheimer’s disease, and traumatic brain injury). Hundreds of studies, including several randomized and controlled trials, have demonstrated efficacy even better than drugs for intractable epilepsy. In general, approximately 50% of children started on the ketogenic diet will have at least a 50% reduction in seizures, with 15% total becoming seizure-free. Many children can reduce or even stop concurrent anticonvulsant drugs after starting the diet.

The ketogenic diet is typically started in the hospital following a brief fasting period, but many centers worldwide have altered this protocol and even “alternative” diets such as the modified Atkins diet have led to flexibility and easier use. These diets can be started at home, without a fast, and sometimes with limited medical supervision. Side effects of the diet do exist but are typically preventable by dietitians and vitamin supplementation. They include constipation, acidosis, hypoglycemia, growth disturbance, elevated serum cholesterol, and kidney stones. Continued use of dietary therapy in Italy and worldwide is expected and will lead to help for children with even the most difficult-to-control seizures.

## A15 Osteomyelitis in children

### Andrzej Krzysztofiak, Elena Bozzola, Laura Lancella, Alessandra Marchesi, Alberto Villani

#### Pediatric Department University–Hospital, Bambino Gesù Children Hospital, Rome, Italy

##### **Correspondence:** Andrzej Krzysztofiak (andrzej.krzysztofiak@opbg.net)

Osteomyelitis (OM) is a bone marrow infection usually caused by bacterial agents. OM is generally caused by haematogenous spread of the infection. It may be rarely secondary to penetrating trauma, surgery or infection in a contiguous site. OM may be classified as acute, sub-acute or chronic infection. Acute haematogenous osteomyelitis (AHO) typically involves the long tubular bones, generally femur, tibia or humerus. *Staphylococcus Aureus* causes 70–90% of AHO in the paediatric age. Methicillin-resistant *Staphylococcus Aureus* (MRSA) prevalence has increased globally (1). Other frequent etiological agents are *Streptococcus pyogenes*, *Streptococcus pneumoniae*, *Group B streptococci* and *Kingella Kingae* (2). Due to immunization policies, the incidence of OM caused by *Haemophilus influenzae* has been significantly decreased in the industrialized States.

Most children affected by AHO have a prolonged bone pain, increased values of erythrocyte sedimentation rate, of C-reactive protein and of white blood cells. (3) The suggested imaging techniques are: radiographic imaging, bone scintigraphy, computed tomography and magnetic resonance imaging (MRI). Radiographic studies are necessary to exclude other bone pathologies which can simulate AHO, such as fracture or tumors (1). Generally, 10-12 days after bone pain onset, osteolytic lesions may be detached on radiographic images. MRI is the most sensitive and specific imaging modality and contributes to an early and prompt of OM. Cultures, bone biopsy and molecular diagnosis should be useful to detach the causative agents.

The therapy of acute osteomyelitis is generally empiric, until the identification of the causative agent. The prescribed antibiotic should have both a good absorption and bone penetration. Treatment with antistaphylococcal penicillin or cephalosporin is effective and safe. If more than 10% of MRSA agents are reported, empiric therapy with vancomycin or linezolid should be prescribed. If the causative agent is detached, empiric therapy should be modified according to the resistance pattern of the organism. Therapy for AHO generally last for 3 to 6 weeks. If a septic arthritis complicates OM, a longer course probably is necessary. Studies on AHO management are limited and guidelines on when to change from parenteral to oral therapy are not yet available. Consequently, standardized approved recommendations on OM are not yet available.

References

1. Harik NS, Smeltzer MS. Management of acute hematogenous osteomyelitis in children. Expert Rev Anti Infect Ther. 2010; 8(2): 175–181.

2. Arnold JC, Bradley JS. Osteoarticular Infections in Children. Infect Dis Clin North Am. 2015 Sep;29(3):557-74.

3. Pääkkönen M, Peltola H. Acute Osteomyelitis in Children. N Engl J Med 2014;370:352-60**.**

## A16 Meeting the expert: is it possible to control procedural pain in newborn?

### Paola Lago^1^, Elisabetta Garetti ^2^, Anna Pirelli ^3^

#### ^1^Paola Lago, NICU, Women’s and Children’s Health department, Azienda Ospedaliera- University of Padua, Padua Italy; ^2^Elisabetta Garetti, NICU, Women’s and Child’s Health Department, Azienda Ospedaliera-University of Modena, Modena Italy; ^3^Anna Pirelli, NICU MBBM Foundation, San Gerardo Hospital, Monza, Italy

##### **Correspondence:** Paola Lago (paolalago9@gmail.com)

**Background**

As soon as they are born, infants in intensive care or in nursery are exposed to painful procedures [1]. There is scientific evidence of this exposure potentially affecting the infants’ pain perception later on, and impairing their neurodevelopmental outcomes in terms of cognition, motor function and brain development [2]. Pain and stress caused by invasive procedures can be prevented or controlled effectively with non-pharmacological and pharmacological interventions, possibly ameliorating the wellbeing of the newborn and their caregivers [3].

**Material and methods**

On behalf of the Italian Neonatology Society’s pain study group, a panel of experts on neonatal pain management gathered all the latest published evidence on the efficacy of analgesic practices for single invasive procedures and applied the GRADE method to reach a consensus on the level of evidence and grade of recommendation for each effective intervention for acute procedural pain control. The best practices for single invasive procedures were classified as environmental, non-pharmacological and pharmacological interventions. The main objective was to update clinicians on the efficacy and safety of proper procedural pain and stress management in the newborn.

**Results**

Strong recommendations with moderate levels of evidence emerged for the use of sweet solutions combined with non-nutritional sucking or other interventions (breastfeeding, skin-to-skin contact, sensorial saturation) during skin-puncturing procedures, which must become standard practice.

Premedication during tracheal intubation is strongly recommended except in emergencies in the delivery room or after acute deterioration. Rapid-onset, short-lived medication is recommended. For mechanical ventilation, there is a strong recommendation and moderate level of evidence for opioid use, not routinely but on an individual basis, in intermittent and/or continuous infusions using the minimal effective dose. Fentanyl seems to be tolerated better than morphine and is recommended in very preterm infants (born under 28 weeks of gestation) at least. For postoperative pain, using paracetamol can spare patients the effects of cumulative doses of opioids for pain control and is recommended. Constant pain monitoring is mandatory to better customize pain treatment.

**Conclusions**

There is evidence of an effective, integrated analgesic approach during invasive and painful procedures in the newborn reducing pain scores and physiological derangements following nociceptive stimuli, and facilitating and expediting the procedure. It is important to customize analgesic treatments, however, and to assess pain routinely with validated pain scales.

**Acknowledgements**

We thank Gina Ancora, Carlo V. Bellieni, Daniele Merazzi, Patrizia Savant Levet, Luisa Pieragostini on behalf of Pain Study Group of Italian Society of Neonatology for their contribution in revising the recently published Guideline on Pain control and prevention during invasive procedure in newborn.

**References**

1. Carbajal R, Rousset A, Danan C, Coquery S, et al. Epidemiology and treatment of painful procedures in neonates in intensive care units. JAMA. 2008;300:60-70.

2. Vinall J, Miller SP, Bjornson BH, Fitzpatrick KP, et al. Invasive procedures in preterm children: brain and cognitive development at school age. Pediatrics. 2014;133:412-21.

3. Lago P, Garetti E, Pirelli A, Merazzi D, Savant Levet P, Bellieni CV, Pieragostini L, Ancora G. Linee Guida per la prevenzione ed il trattamento del dolore nel neonato. Milano:Biomedia;2016.

## A17 Topical intranasal bacteriotherapy: experience in acute otitis media

### Paola Marchisio^1^, Maria Santagati^2^, Stefania Stefani^2^, Susanna Esposito^1^, Nicola Principi^1^

#### ^1^Pediatric Highly Intensive Care Unit, Department of Pathophysiology and Transplantation, Università degli Studi di Milano, Fondazione IRCCS Ca’ Granda Ospedale Maggiore Policlinico, Milan, Italy; ^2^Department of Biomedical and Biotechnological Sciences, MMAR Laboratory, University of Catania, Catania, Italy

The prevention of acute otitis media (AOM) is currently one of the primary goals of paediatric care. This is mainly true for recurrent episodes, but can also be considered in relation to avoiding a first episode in otherwise healthy children. AOM is a multifactorial disease, favoured by many predisposing factors. It usually follows a viral infection of the upper respiratory tract: therefore prevention relies on reducing risk factors, viral respiratory infections, and nasopharyngeal bacterial colonisation.

Reducing environmental risk factors (e.g. day care attendance, passive smoking, pollution), favoring protective factors (e.g. breastfeeding, hand hygiene), prolonged low-dosage antibiotics, immunoprophylaxis with influenza and conjugate pneumococcal vaccine, vitamin D supplementation, probiotics, adenoidectomy, tubes placement, and alternative medicine have all been proposed as prophylactic measures. However, none has been demonstrated to be able to complete solve the problem of recurrent AOM: recurrence in the treated children is usually reduced in comparison with that of controls, but, even when various preventive treatments are used at the same time, a relevant number of children continue to have AOM. In addition, there are concerns for some of these measures. Antibiotic prophylaxis regimen is associated with an increased risk of side effects and the emergence of resistant bacteria. Moreover, safety and tolerability of most alternative medicine remedies are not precisely defined [1,2].

In the ‘90s it was evidenced that AOM could more easily occur when commensal saprophytic flora of the nasopharynx was reduced, causing a relevant proliferation of the asymptomatically carried otopathogens [3]. Topical nasal administration of probiotics was considered as a method to reduce the risk of recurrent AOM in children. The most largely studied microorganism was α-hemolytic *Streptococcus* (AHS). Unfortunately, results were discordant and, after the positive study of Roos et al [4], the method was not developed mainly because its safety was questioned. With time, the potential pathogenic role of the bacteria that normally colonize human nasopharynx early in life has been clarified. *Streptococcus salivarius 24SMB* has been identified as an oral probiotic, characterized by good safety, ability to inhibit otopathogens responsible of AOM, and the absence of virulence and antibiotic resistance genes.

In a prospective, randomized, double-blind, placebo controlled study of intranasal administration of *S. salivarius 24SMB* in children with a history of recurrent AOM, the number of patients who did not experience any further episode during the study period as well as the mean number of AOM episodes were significantly lower in patients in whom colonization by *S. salivarius 24SMB* was demonstrated than in those treated with *S. salivarius 24SMB* for whom no colonization was observed [4].

The study supports the potential ability of *S. salivarius 24SMB* administered intranasally in reducing the risk of AOM in otitis-prone children. Ongoing studies are in progress to confirm these data in larger populations and identify the factors that in some children do not allow *S. salivarius 24SMB* colonization.

**References**

1. Marchisio P, Nazzari E, Torretta S, Esposito S, Principi N. Medical prevention of recurrent acute otitis media: an updated overview, Expert Rev Anti Infect Ther 2014; 12: 611-620.

2. Marom T, Marchisio P, Tamir SO, Torretta S, Gavriel H, Esposito S. Complementary and Alternative Medicine Treatment Options for Otitis Media: A Systematic Review. Medicine (Baltimore). 2016;95:e2695.

3. Marchisio P, Claut L, Rognoni A, et al. Differences in nasopharyngeal bacterial flora in children with non-severe recurrent acute otitis media and chronic otitis media with effusion: implications for management. Pediatr Infect Dis J 2003;22:262-268

4. Roos K, Håkansson EG, Holm S. Effect of recolonisation with “interfering” alpha streptococci on recurrences of acute and secretory otitis media in children: randomised placebo controlled trial. Brit Med J 2001; 322:210–212.

5. Marchisio P, Santagati M, Scillato M, et al. Streptococcus salivarius 24SMB administered by nasal spray for the prevention of acute otitis media in otitis-prone children. Eur J Clin Microbiol Infect Dis. 2015; 34:2377-2383.

## A18 Children with rare disease in Emergency Department

### Valeria d’Apolito^1^_,_ Luigi Memo^2^, Angelo Selicorni^3^

#### ^1^Pediatric Department, Fondazione MBBM, S. Gerardo Hospital, Monza, Italy; ^2^Pediatric Department, S. Martino Hospital, Belluno, Italy; ^3^Pediatric Department, S. Anna Hospital, Como, Italy

##### **Correspondence:** Luigi Memo (luigi.memo@tin.it)

**Background**

Children With Special Health Care Needs (CSHCN) represent an important population from health and economic policy prospective. They are at heightened risk for having acute illness, related to chronic health conditions or not, and frequently attend the Emergency Department (ED). Acute emergency situations of CSHCN are an important challenge for pediatrician.

**Objective**

The primary objective of this study was to describe what happens when these children go to ED, we described features of their admissions to ED (why, where and when) to estimate the intensity of care they need and receive there.

**Methods**

We described 2897 Emergency department admissions of CSHCN occurred in 60 hospitals of different levels in Italy since 1 December 2015 until 31 may 2016. For each admission we described time it occurred, level of hospital, children condition, the reason for accessing ED, triage code, the medical case management (examinations, consultations, treatments) and the outcome.

**Results**

55% of the 2897 children had a genetic syndrome, 21% of our population used some device, 25% of them has almost two devices. 57% of the admissions occurred in a tertiary hospital. The most common reason was respiratory symptoms, the second one was fever. 4% of children needed ED because of device malfunctioning. We compared the rates of red and yellow triage codes in our population with data of general Italian pediatric population of the Italian Society of emergency medicine and pediatric urgency. We observed that in our population red and yellow triage tag were respectively ten *times* and five times higher than in *general Italian pediatric population*. Tertiary hospital accepted the highest number of patients with severe symptoms. Concerning to cases management data showed that 72% of our patients underwent almost one examination. About the outcome of patients our data revealed that 42% of them wasn’t discharged. We compared this number with the hospitalization rate of general pediatric Italian population during year 2011, and we observed that percent of hospitalization we observed is about six times higher than in *general Italian pediatric population*.

**Conclusion**

CSHCN present often symptoms more severe than general pediatric population. These patients frequently require higher level care and have higher percentage of hospitalization [1]. Because about 40% of them arrives to ED of a not specialized hospital, all physicians have to be aware of these diseases because if you know them you can take care of them better.

**References**

1. Minasi D, Pitrolo E, Paravati F. L’appropriatezza dei ricoveri ospedalieri in età pediatrica in Italia. Area Pediatrica. 2014; 15:13-16.

## A19 Role for probiotics in intestinal dysbiosis of the infant

### Vito Leonardo Miniello, Lucia Diaferio

#### Department of Pediatrics, “Aldo Moro” University of Bari, Giovanni XXIII Hospital, Bari 70126, Italy

##### **Correspondence:** Vito Leonardo Miniello (vito.miniello@libero.it)

The digestive tract is a complex ecosystem in which microbial communities (gut microbiota) interact with each other and with their host. Infancy is a critical stage for the foundation and development of the intestinal microbiota. During vaginal delivery bacterial exposure from the birth canal is a pivotal precursor for the colonisation of the infant gut in the first few days of life [1]. The initial microbial colonization is a stepwise process and interactions between the colonizing bacteria and the human host ultimately have a key influence on health and disease [2]. From birth, the normal gut microbiota contributes to the development of gut functions, provides protection against infections, contributes to the regulation and maintenance of intestinal barrier function, establishes immune and metabolic homeostasis later in life, and promotes tolerance of foods. The neonatal colonization pattern is markedly influenced by several perinatal environmental factors such as the mode (vaginal *vs* caesarean) and the place (home-born *vs* hospital-born) of delivery, the maternal microbiome, the number of siblings, infant feeding (breast milk *vs* infant formula), perinatal drug-based therapies (antibiotics), timing and composition of weaning, and maternal infections [3]. Despite the fact that most of the causality is not yet fully understood, shift in the commensal gut microbial communities with implication to disease is often referred to as dysbiosis. Various short- and long term chronic inflammatory disorders can be explained in part by disturbed immune and metabolic functions induced by the aberrant microbial colonization [4]. It has been suggested that early infancy gut microbial alteration could influence metabolic health of children and adolescents. Increased interest in the effects of the intestinal microbiota on human health has resulted in attempts to optimize the microbial ecosystem by the so called ‘gut microbiota biomodulators’ [5], such as probiotics, prebiotics, synbiotics or postbiotics. Probiotics are “live micro-organisms which when administered in adequate amounts confer a specific health benefit on the host” [6]. In infants with inadequate or abnormal early intestinal colonization (dysbiosis), whether induced by Caesarean section, premature delivery or excessive use of perinatal antibiotics, probiotics might prevent metabolic and immuno-dysregulation by exerting anti-inflammatory effects, improving intestinal function barrier and modulating immune responses. In-vitro and animal studies have generated most of the mechanistic rationale for the use of probiotics that act through a number of different pathways [7-10]. Noteworthy, the ability of probiotics to influence immune and metabolic pathways differs greatly depending on the strain in question.

**References**

1. Dominguez-Bello MG, Costello EK, Contreras M, et al. Delivery mode shapes the acquisition and structure of the initial microbiota across multiple body habitats in newborns. Proc Natl Acad Sci USA. 2010; 107: 11971–5.

2. Rautava S, Luoto R, Salminen S, et al. Microbial contact during pregnancy, intestinal colonization and human disease. Nat Rev Gastroenterol Hepatol. 2012; 9: 565-7.

3 .Penders J, Thijs C, Vink C, et al. Factors influencing the composition of the intestinal microbiota in early infancy. Pediatrics. 2006; 118: 511–21.

4. Carding S, Verbeke K, Vipond DT, et al. Dysbiosis of the gut microbiota in disease. Microb Ecol Health Dis. 2015; 26: 26191.

5. Miniello VL Colasanto A, Diaferio L, et al. Gut microbiota biomodulators: when the stork comes by the scalpel. Clin Chim Acta. 2015; 451: 88-96.

6. Food and Agriculture Organization/World Health Organization. Joint FAO/WHO expert consultation on evaluation of health and nutritional properties of probiotics in food including powder milk with live lactic acid bacteria.

7. Prescott SL, Björkstén B. Probiotics for the prevention or treatment of allergic diseases. J Allergy Clin Immunol. 2007; 120: 255–62.

8. Gill HS, Rutherfurd KJ, Cross ML, et al. Enhancement of immunity in the elderly by dietary supplementation with the probiotic Bifidobacterium lactis HN019. Am J Clin Nutr. 2001; 74: 833–83.

9. Yang F, Wang A, Zeng X, et al. Lactobacillus reuteri I5007 modulates tight junction protein expression in IPEC-J2 cells with LPS stimulation and in newborn piglets under normal conditions. BMC Microbiol. 2015;15:32.

10. Patel RM, Myers LS, Kurundkar AR, et al. Probiotic bacteria induce maturation of intestinal claudin 3 expression and barrier function. Am J Pathol. 2012; 180: 626–35.

## A20 Apparent life threatening events (ALTE) in daily clinical practice

### Antonella Palmieri (antonellapalmieri@gaslini.org)

#### Chief of SIDS/ALTE Liguria Center, Pediatric Emergency Department, Giannina Gaslini Children’s Hospital, Genoa, Italy

**Background**

What is ALTE? Apparent life threatening events (ALTE) is an acronym to indicate the presence of a series of alarming symptoms in newborn and infants (such as apnea, change in color or muscle tone, coughing, gagging, transient impairment of consciousness) that are recounted or experienced by parents or relatives. Rarely the doctor, trusted by parents for patient management, is a witness of the facts. Symptoms often occur during sleep, and the anxiously parents can alter the report of events. This fact makes difficult and ethically complex the management of young patients.

It’s estimated that the percentage of ALTE’s cases is about 2% of the total number of accesses to the Emergency room (ER): there are few cases that require a multidisciplinary approach and specific guidelines.

**Materials and methods**

In one year, our Ligurian Center performs about 600 follow-up visits and about 60-70 hospitalizations for cases of ALTE. The 98% of cases that come to the paediatric emergency departments is subjected to hospitalization.

The remaining cases with minimal events, which do not include all the clinical criteria but which have generated anxiety in parents, are admitted to “Short-stay Observation".

All patients are subjected to first-level exams, culture tests and ECG; 98% also perform EEG and transfontanellar ultrasound. During hospitalization or later in the follow-up, second-level exams are programmed according to the specialists. For about a year, with the collaboration with the Clinical Genetics Centre, in selected patients, and in cases of idiopathic ALTE we have launched investigations directed to the search of PHOX2B. A very select part of the patients undergoes home cardiac monitoring.

**Results**

The Follow-up is completed by 95% of patients while in 5% it’s interrupted for family rejection or due to the fact that the child comes from other regions, and he’s sent to nearest reference centers.

For the management of ALTE events is not only important the clinical approach but also the communication with parents, the CPR training and the use of cardio-monitor.

**Conclusion**

The compilation of guidelines is important for the creation of shared criteria for patient management both in acute and during hospitalization, by programming first and second level examinations; it helps to understand the need for a regional network of reference in close collaboration with regional reference center to optimize the management of patients with ALTE [1].

**References**

1. Mittal MK, Sun G, Baren JM. A clinical decision rule to identify infants with apparent life-threatening event who can be safely discharged from the emergency department. Pediatr Emer Care. 2012;28:1-7.

## A21 How are we treating infants with bronchiolitis?

### Luciana Parola (luciana.parola@asst-ovestmi.it)

#### Department of Pediatrics, Neonatology and Neonatal Pathology, Hospital “G.Fornaroli”, ASST Ovest Milanese, via Al Donatore di Sangue 50, 20013 Magenta (Milan), Italy

**Background**

Bronchiolitis is a common and potentially severe disease in infants. Many guidelines are available about its management. Since there is a great variability on diagnosis and treatment, a systematic data collection is necessary. The “Network for Bronchiolitis” was created by the “Accreditation and Quality Improvement Working Group” of the Italian Society of Pediatrics to evaluate the level of implementation of local guidelines [1, 2]. Other objectives of the network were: scientific research, surveillance of rare events, supervising of complex phenomenon, standardization of diagnostic criteria and therapeutic processes, generation of an hospital network able to allow a fast and profitable data exchanges. The subscriptions on the network permitted also to all the participants to monitor their own results related with national and regional data.

**Materials and methods**

In this study all patients less than two years old admitted to acute bronchiolitis were included. Each data was recorded after discharge from a single operator of each hospital participant and loaded into an anonymous electronic report form, created in collaboration with the Subspecialty Scientific Societies and available in a specific website.

**Results**

Data were collected between the 1st of October 2014 until the 30th of July 2016. Ten centers participated in the study and a total of 761 cases of bronchiolitis were collected (Table 3).

62% of the patients arrived spontaneously to the hospital, and 26% was referred from the general practitioner; 83% of all were born at term. The examinations done during admission are listed in Table 4 and the treatments in Table 5.

The risk factors revealed with more frequency were hospital discharge in epidemic period (8%) and older siblings (14%). The more important discordances from local guidelines were: chest x ray (done in 45% of the cases), administration of antibiotics (50%) and steroids (49%). However, the use of chest x ray progressively decreased during years of survey (33% on 2016).

**Conclusions**

The use of networks is very useful and practice to verify the applications of guidelines in clinical practice. Despite many expert recommendations and guidelines, we found lot of inappropriate diagnostic tests and treatments in patients admitted with bronchiolitis. As reported in the literature [3], the participation in a network contributed to an important reduction of inappropriate therapy and diagnostic tests in bronchiolitis. Educational approaches could also lead to more improvements.

**Acknowledgements**

Parisi Giuseppe Presidio Ospedaliero "Anna Rizzoli" Ospedale ASL

Bruni Paola A.O. di Circolo di Melegnano - P.O. Vizzolo Predabissi Azienda Ospedaliera

Esposito Susanna, Tagliabue Claudia Fondazione IRCCS Ca' Granda Ospedale Maggiore Policlinico

Flores d'Arcais Alberto Ospedale Civile di Legnano

Kantar Ahmad Policlinico San Pietro Struttura Privata Accreditata

Longhi Riccardo, Ortisi Maria Teresa Ospedale Sant'Anna Como

Montrasio Giovanni Ospedale di Circolo Busto Arsizio-Presidio di Saronno

Parola Luciana, Racchi Elisabetta Ospedale "G.Fornaroli" Magenta

Rondanini Gian Filippo, Calzi Patrizia AO Desio e Vimercate (poc Vimercate)

Bellettato Massimo Azienda Ospedaliera San Bortolo

**References**

1. Baraldi,E. Lanari M., Manzoni P. et al. Inter-society consensus document on treatment and prevention of bronchiolitis in newborns and infants. Ital J Pediat. 2014, 40:65.

2. Ralston S.L., Lieberthal A.S., Meissner H.C. et al. Clinical practice guideline: the diagnosis, management, and prevention of bronchiolitis. Pediatrics. 2014; 134: e1474-e1572.

3. Ralston S.L., Garber M.D., Rice-Conboy E. et al. A multicenter collaborative to reduce unnecessary care in inpatient bronchiolitis. Pediatrics. 2016; 137: e20150851.

**Table 3 (abstract A21).** Characteristics of patients admitted to bronchiolitis

Gender	Male	441 (57.9%)
	Female	320 (42.1%)
Age at admission	< 1 month	100 (13.1%)
	> 1 to ≤ 3 months	301 (39.5%)
	> 3 to ≤ 6 months	210 (27.6%)
	> 6 to ≤ 12 months	139 (18.3%)
	> 12 months	11 (1.5%)
Month of admission	October	22 (2.9%)
	November	66 (8.7%)
	December	255 (33.5%)
	January	195 (25.6%)
	February	108 (14.2%)
	March	57 (7.5%)
	April	30 (3.9%)
	Other Months	28 (3.7%)

**Table 4 (abstract A21).** Examinations done during admissions

Chest X Ray	339 (44.5%)
Blood Gas Analysis	459 (60.3%)
Blood Test: White Cells Count/C Reactive Protein	726 (95.4%)
Blood Saturation Monitoring	706 (92.7%)
Positive RSV	433 (56.9%)
Negative RSV	313 (41.1%)
Search For Other Etiological Causes	224 (29.4%)

**Table 5 (abstract A21).** Treatment administered during admissions

Inhalatorial Adrenaline	Yes	157 (20.6%)
	No	589 (77.4%)
	Data not available	15 (2.0%)
Antibiotics	Yes	381 (50.1%)
	No	366 (48.1%)
	Data not available	14 (1.8%)
Inalhatory Beta Agonists	Yes > 24 hours	447 (58.7%)
	Yes ≤ 24 hours	29 (3.8%)
	No	270 (35.5%)
	Data not available	15 (2.0%)
Intravenous Hydratation	Yes	259 (34.0%)
	No	490 (64.4%)
	Data not available	12 (1.6%)
Oxygen Administration	Yes	465 (61.1%)
	No	289 (38.0%)
	Data not available	7 (0.9%)
Inalhatorial Hypertonic	Yes total	387 (50.8%)
	YES (not known the percentage)	192 (25.2%)
	Yes at 3%	195 (25.6%)
	Yes at 5%	0 (0.0%)
	No	369 (48.5%)
	Data not available	5 (0.7%)
Steroids	Yes	375 (49.3%)
	No	370 (48.6%)
	Data not available	16 (2.1%)

## A22 Benign Familial Macrocephaly/Megalencephaly, does it really exist?

### Ettore Piro (ettore.piro@unipa.it)

#### Department of Science for Health Promotion and Mother and Child Care “G. D'Alessandro” University of Palermo, Palermo, Italy

Macrocephaly is defined as an occipitofrontal circumference (OFC) greater than two standard deviations (SD) above the mean for a given age, sex, and gestational age (i.e., ≥97th percentile), and can be related to an increased volume of one of the four components; brain parenchyma or megalencephaly, cerebrospinal fluid, blood, and thickening of cranial bones.

Megalencephaly can be due to anatomic or metabolic conditions. Anatomic megalencephaly is caused by an increase in the size or number of brain cells [1].

The most common type of anatomic megalencephaly is Benign Familial Megalencephaly (BFM).

In BFM, increased OFC, usually present at birth, is clearly evident in the first months of life, increasing to greater than the 90th percentile, typically 2 to 4 cm above, but parallel to, the 98th percentile. OFC may increase by 0.6 to 1 cm per week (compared with the normal 0.4 cm/week) [2]. Head growth velocity slows to a normal rate by approximately six months of age.

BFM condition in OMIM is termed Benign Familial Macrocephaly (# 153470), strong family history of isolated macrocephaly is frequent, mainly in male. The genetic basis for this nonsyndromic macrocephaly is multifactorial with a polymorphic genetic basis, the risk of recurrence appears to be much lower than it would be on the assumption of autosomal dominant inheritance, as previously supposed. The benignity of BFM is related to the normal long term outcome of global psychomotor development.

Recently progresses in molecular genetic studies of brain development, focusing on rare congenital conditions associated with megalencephaly, have identified an important role of genes and relatives products as MLC1 (OMIM 605908), that encodes a transmembrane protein that associates with the Na,K-ATPase beta-1 subunit and the hepatic and glial cell adhesion molecule HEPACAM/GLIALCAM (OMIM # 611642).

MLC1 is related to the majority of cases of Megalencephalic Leukoencephalopathy with Subcortical Cysts (MLC, OMIM # 604004). MLC1 is an oligomeric membrane protein that is expressed almost exclusively in the brain. GlialCAM acts as a MLC1 beta subunit needed for its correct trafficking to cell junctions. The HEPACAM mutations are either recessive or dominant with different pathogenic effects depending on the cellular region involved. Recessive mutations are identified in MLC patients without MLC1 mutations, while in 60% of the families with dominant HEPACAM mutations, the affected persons display BFM [3].

In the first 2-3 years developmental surveillance and brain imaging of a macrocephalic child are of great importance for making a definitive diagnosis.

**References**

1. Menkes HJ, Sarnat HB. Child Neurology. 6th ed, Lippincot Williams & Wilkins Philadelphia 2000. 354-357.

2. DeMyer W. Megalencephaly: types, clinical syndromes, and management. Pediatr Neurol. 1986; 2: 321-8

3. López-Hernández T, Ridder MC, Montolio M, Capdevila-Nortes X, Polder E, Sirisi S, Duarri A, Schulte U, Fakler B, Nunes V, Scheper GC, Martínez A, Estévez R, van der Knaap MS. Mutant GlialCAM causes megalencephalic leukoencephalopathy with subcortical cysts, benign familial macrocephaly, and macrocephaly with retardation and autism. Am J Hum Genet. 2011.88:422-32.

## A23 Feeding and nutritional issues in children with neurodisability

### Claudio Romano, Maria Ausilia Catena, Sabrina Cardile

#### Unit of Pediatrics, Department of Human Pathology in Adulthood and Childhood “G. Barresi” - University of Messina, Messina, Italy

##### **Correspondence:** Claudio Romano (romanoc@unime.it)

**Background**

Undernutrition, growth failure, overweight, micronutrient deficiencies are common conditions among children with neurological impairment (NI). Nutritional support may restore linear growth, normalize weight, improve quality of life and decrease the frequency of aspiration. The role of a multidisciplinary team is crucial.

**Feeding and nutritional issues**

Feeding difficulties (FD) in children with neurological impairments (NI) are due to an organic cause. Cerebral Palsy (CP) patients constitute the most frequently found group of NI children with dysphagia or feeding difficulties [1]. Inadequate caloric intake is correlated with oral motor dysfunction, inability to self feed, and gastrointestinal disease, such as gastroesophageal reflux disease (GERD) and constipation , as well as respiratory problems [2-3-4-5].

Physical examination can underline signs of malnutrition or micronutrient deficiencies. In the feeding history, type of meal, meal times, child’s position during the meal, child's autonomy and the role of the caregiver, recurrence of specific symptoms during the meal should be taken into account. [6]. Triceps skinfold thickness is the best anthropometric index to assess the nutritional status of these children [7-8]. High incidence of anemia has been found and attributed to secondary iron deficiency [9]. Even non-nutritional factors play a significant role, such as type and severity of neurological disease and antiepileptic drugs use [9]. Nutritional support is essential for the care and quality of life of NI children (indications for artificial nutrition in Table 6). Enteral tube feedings are indicated in children who cannot meet their energy and nutrients needs by oral feeding alone or in children with swallowing dysfunction and risk of aspiration [10]. There are many methods to determine dietary energy needs in NI children (Table 7). Percutaneous endoscopic gastrostomy (PEG) placement is indicated in the case of long-term enteral nutrition (>3 months) as it is more comfortable than a nasogastric tube [11]. In children who do not tolerate gastric feeds, with severe gastroesophageal reflux, risk of aspiration, are poor candidates for fundoplication, can be considered the possibility of using gastrojejunostomy or jejunostomy. There are various methods of feed administration: bolus feeding, intermittent or continuous infusion of formula. Many varieties of commercial enteral formulas are available, with various energy densities. [12].

**Conclusions**

Nutritional care of children with neurodevelopmental disabilities has improved with the advent of various enteral access methods and better tolerated enteral formulas. Nutritional assessment and support must be an integral part of the care of these children with close monitoring and early nutritional intervention.

**References**

1. Dahl M, Thommessen M, Rasmussen M et al. Feeding and nutritional characteristics in children with moderate or severe cerebral palsy. Acta Pediatr. 1996;85:697–701.

2. Benfer KA, Weir KA, Bell KL, et al. Oropharyngeal dysphagia and gross motor skills in children with cerebral palsy. Pediatrics. 2013;131: e1553.

3. Catto-Smith AG, Jimenez S. Morbidity and mortality after percutaneous endoscopic gastrostomy in children with neurological disability. J Gastroenterol Hepatol. 2006;21:734-8.

4. Del Giudice E, Staiano A, Capano G, et al. Gastrointestinal manifestations in children with cerebral palsy. Brain Dev. 1999 ;21:307-11.

5. Veugelers , Benninga MA, Calis EA, et al. Prevalence and clinical presentation of constipation in children with severe generalized cerebral palsy. Dev Med Child Neurol. 2010;52:e216-21.

6. Stevenson RD. Use of segmental measures to estimate stature in children with cerebral palsy. Arch Pediatr Adolesc Med 1995;149:658-662.

7. Gurka MJ, Kuperminc MN, Busby MG et al. Assessment and correction of skinfold thickness equations in estimating body fat in children with cerebral palsy. Dev Med Child Neurol. 2010;52;e35-41.

8. Kuperminc MN, Gurka MJ, Bennis JA, et al. Anthropometric measures: poor predictors of body fat in children with moderate to severe cerebral palsy Dev Med Child Neurol. 2010;52:824-30.

9. Kilpinen-Loisa P, Pihko H, Vesander U et al. Insufficient energy and nutrient intake in children with motor disability. Acta Paediatr. 2009, 98, 1329–1333.

10. Sangermano M, D’Aniello R, Massa G et al. Nutritional problems in children with neuromotor disabilities: An Italian case series. Ital. J. Pediatr. 2014;40:61–65.

11. Papadopoulos A, Ntaios G, Kaifa G et al. Increased incidence of iron deficiency anemia secondary to inadequate iron intake in institutionalized, young patients with cerebral palsy. Int. J. Hematol. 2008;88:495–497.

12. Sánchez-Lastres J, Eiris-Punal J, Otero-Cepeda JL et al. Nutritional status of mentally retarded children in north-west Spain. I.Anthropometric indicators. Acta Paediatr. 2003;92:747–753.

**Table 6 (abstract A23).** Indication for artificial nutrition for NI children

Evidence of oral motor feeding difficulties	
Undernutrition (weight for height < 80% of expected, BMI < 5° percentile)	
Growth failure (height for age < 90% of expected)	
Overweight (BMI > 95° percentile)	
Individual nutrient deficiencies

**Table 7 (abstract A23).** Calculating energy needs of neurologically impaired children

1. Krick method Kcal/day = (BMR x muscle tone factor x activity factor) + growth factor BMR basal metabolic rate (kcal/day) = body surface area (m^2^) x standard metabolic rate (kcal/m^2^/h) x 24 h Muscle tone factor: 0.9 if decreased, 1.0 if normal, 1.1 if increased Activity factor : 1.15 if bedridden, 1.2 if dependant, 1.25 if crawling, 1.3 if ambulatory Growth factor : 5 kcal/g of desired weight gain	
*2. Height-based method* 14.7 cal/cm in children with motor dysfunction 13.9 cal/cm in ambulatory patients with motor dysfunction 11.1 cal/cm in non-ambulatory patients	
3. Resting energy expenditure-based method 1.1 x measured resting energy expenditure	

## A24 The preterm infants and predisposition to post-infectious respiratory diseases

### Oliviero Sacco, Donata Girosi, Roberta Olcese, Mariangela Tosca, Giovanni Arturo Rossi

#### Pediatric Respiratory and Allergy Units, Giannina Gaslini Hospital and Research Institute, Genoa, Italy

##### **Correspondence:** Giovanni Arturo Rossi (giovannirossi@ospedale-gaslini.ge.it)

A growing body of literature has documented that, as compared with term infants, preterm infants are at greater risk to develop a variety of medical complications. With regard to the respiratory system all forms of morbidity, including respiratory distress syndrome, transient tachypnea of the newborn and pulmonary hypertension, affect preterm infants at a higher rate than infants of more advanced gestational age (GA). In addition, low GA represents in the first months of life a major risk factor for hospitalization for bronchiolitis, the first viral lower respiratory tract infection (LRTI). Results of a recent review suggested that many adverse respiratory consequences of the first viral infection in preterm infants are likely the result of persistent modifications of the pulmonary structures. However, as demonstrated for severe respiratory syncytial virus (RSV)-induced bronchiolitis, functional abnormalities of the airway reactivity and of the immune system function, can also play a significant role. The adaptive immune response to RSV infection is greatly attenuated in preterm infants, with delayed virus clearance, increased damage to the airway structures, induction of a detrimental Th2 response and of a Th2 immune memory. In addition to promote lung injury, a non effective immune response facilitates the recurrence of severe symptoms with subsequent exposure to RSV, but also to other pathogens or to pollutants. Early-life severe RSV-induced LRTI also induces an abnormal neural control of the bronchial structures, resulting in airway hyperreactivity and in amplification of the local inflammatory reaction. The “RSV-induced neurogenic inflammation” appears to potentiate the cholinergic and excitatory noncholinergic, nonadrenergic neural pathways that favors bronchoconstriction, but also enhances mucus production and increases vascular permeability. These changes in the inflammatory and immune response and in the sensory and motor nerve reactivity are deemed to play a short- and long-term significant role in the increased predisposition to post-infectious respiratory diseases. Indeed, after hospitalizations for RSV-induced LRTI, preterm infants experience higher re-hospitalization rates, longer hospital stays, and more frequent outpatient visits as compared with infants of similar GA who were not hospitalized for RSV. In addition, RSV-induced LRTI has been also associated with an increased risk of reduced lung function and irreversible airway obstruction up to the age of 18-31 years. An effective prevention and/or treatment strategies are needed to protect premature infants from severe respiratory infections in early life but also to reduce the predisposition to post-infectious respiratory diseases in childhood and adulthood.

## A25 Radiation protection in pediatric age: current laws and patients and relatives information

### Sergio Salerno, Maria Chiara Terranova

#### Dipartimento di Biopatologia e biotecnologie mediche, Università di Palermo, Palermo, Italy

##### **Correspondence:** Sergio Salerno (sergio.salerno@unipa.it)

Patients communication of radiation risk is mandatory, as underlined by the new European Community directives (COUNCIL DIRECTIVE 2013/59/EURATOM of 5 December 2013 laying down basic safety standards for protection against the dangers arising from exposure to ionizing radiation, repealing Directives 89/618/Euratom, 90/641/Euratom, 96/29/Euratom, 97/43/Euratom, 2003/122/Euratom)[1]. They emphasize the need for justification of medical exposure, and should strengthen the requirements concerning information to be provided to patients, the recording/reporting of doses from medical procedures- “dose bill”- the use of diagnostic reference levels and the availability of dose-indicating devices, considering that information about patients exposure becomes now a part of medical records [1].

The Directives define also practitioner’s “clinical responsibility” for individual medical exposure: justification; optimization; clinical evaluation of outcomes; cooperation with other specialists and staff regarding practical aspects of medical radiological procedures.

All these points become critical in pediatric care as children are more sensitive to ionizing radiations and have longer life expectation than adults [2].

So relatives’ or legal guardians’ informed consent - mandatory for any radiological procedure - becomes crucial and delicate.

Keyword is communication:

Even if the risks of exposure to ionizing radiations are widely known, the great effect variability due to dose, sex, age, type of exam and different irradiated body parts make univocal risk settlement and comprehension complicated.

WHAT to communicate: in daily routine we use Quantitative Index (CTDI-vol, DLP) -but they are poorly understood by parents - or Equivalent Doses (number of Chest X-Rays; time to receive the same dose from natural background radiations)- that may vary widely or may be perceived as number of months/years of life that would be lost undergoing radiological exam.

HOW to communicate: we can report the cancer-developing risk as possible collateral damage from radiological approach, expressed as increased percentage compared with population. Practitioners should avoid catastrophic data (“The risk for your newborn of having a radiation-induced cancer due to pelvic CT is DOUBLE: 0.3% plus 0.3%”), preferring less mistakable information (“the probability for your child of having a normal infancy is 96.4%, almost the same of non-exposed children: 96.7%”) [3].

Informed consent in pediatric radiology has a deeper impact, not only because of the increased susceptibility to ionizing radiation in childhood, but also because of parents’ emotional implications.

That’s the reason why all practitioners, especially those who care children, should have an adequate education in Radiation Protection, in order to be able to assess and communicate risks/benefits of radiological procedures.

**References**

1. COUNCIL DIRECTIVE 2013/59/EURATOM - 5 December 2013

2. Salerno S and Geraci C. Radiation Protection in pediatrics age. Italian Journal of Pediatrics 2014;40 (Suppl 1): A62

3. Wagner LK. Toward a holistic approach in the presentation of benefits and risks of medical radiation. Locke PA, ed. 46th annual meeting of the National Council on Radiation Protection. Bethesda, MD: National Council on Radiation Protection, 2010:22–23

## A26 How to reply to revisors: advice and something else

### Francesca Santamaria (santamar@unina.it)

#### Dipartimento di Scienze Mediche Traslazionali, Università Federico II Napoli, Naples, Italy

“Peer reviewers” or “referees” are experts who are requested to assess critically a manuscript that has been submitted for publication. An essential recommendation, even to the most expert authors, is take the role of the referee to try to be as impartial as possible towards their own paper.

Once acquired the concept that for good writing you need “edit, edit, and edit” your manuscript, few, additional simple advices before the first submission are:

- Do not apology for not doing investigation correctly: *reviewers know that this is not a good excuse.*
- Consider that non significant results cannot misquoted as trends: r*eviewers are fully aware of this.*
- Ensure that the manuscript communicates information clearly: *a reviewer is also a reader.*
- Do not lengthen the discussion: this will hardly distract the reviewer’s attention.
- Do not disregard to get your manuscript checked by an English-speaking author. Referees pay much attention to typos or poor spelling or grammar.

While preparing the revised paper, first of all keep in mind that criticisms should be read and discussed by all authors for being sure that you all fully understood the meaning of the observations. While preparing the point-by-point response, do not forget to specify all the changes you made, including the lines and pages of the revised version of the manuscript that contain them. Sometimes, reviewers’ questions are unclear: if this is the case, do not hesitate to ask for further clarification. Provide an updated list of references: new studies might have been published since your original drawing up. Finally, pay attention to the tone of your reply: be courteous and pleasant, and do not omit to thank both editors and referees for their help in improving your paper.

Should you follow all these suggestions, you would also contribute to make the reviewers life easy. Now, it’s time to resubmit a revised version of the paper and expect that it will be hopefully selected for publication.

## A27 Children affected with rare diseases without a diagnosis

### Angelo Selicorni^1,2^, Giorgia Mancano^1,2^, Silvia Maitz^2^

#### ^1^UOC di Pediatria, ASST Lariana, Como, Italy; ^2^UOS Genetica Clinica Pediatrica, Clinica Pediatrica, Fondazione MBBM, Monza, Italy

##### **Correspondence:** Angelo Selicorni (angelo.selicorni61@gmail.com)

Even though our ability to recognize very rare phenotypes is improving, thousands of patients are still undiagnosed despite multiple proper diagnostic evaluations and testing. From a patient’s point of view, living without a diagnosis means living without a name, without genetic tests in order to perform a proper genetic counseling, without prognosis, without hope for an active research program and future treatment.

Diagnostic use of microarray technology in recent years succeeded in improving the possibility of defining genetic basis of very complex but unclassified phenotypes. Moreover the new utilization of Next Generation Sequencing (NGS) technology showed its high potentiality in discovering new disease-related genes and/or mutations in an already known gene with a very atypical phenotype. This is what happened, for example, in the Canadian national project (FORGE) in which 362 families have been studied with a Whole Exome Sequencing (WES) approach showing a detection rate of 51,7% (188 families characterized). In the same program, a mutation in a possible pathogenetic gene has been identified in 28 families, even though data were not enough to be conclusive yet [1]. A large and increasing number of reports have been published in the last years with different detection rates, based on the population which underwent the molecular study. This new approach seems to be also cost effective as showed by Valencia et al [2] who calculated the high number of tests performed by each of their patients before having a WES test. For this reason, Shashi et al [3] suggested to adopt WES as a test to be used as first/second line approach when phenotype is not so typical and the gestalt fails to guide the diagnostic process.

In this direction, since April 2016, Telethon Foundation is funding the Telethon Undiagnosed Diseases Program (TUDP), an intramural research project in which TIGEM lab, in cooperation with OPBG (Ospedale Pediatrico Bambino Gesù) lab, will test 300-350 families coming from a very deep clinical selection performed by three clinical centers. All pediatricians and all specialists can submit their patients to TUDP by filling a quite simple web form on the institutional Telethon web site (Malattie Senza Diagnosi, http://www.telethon.it/cosa-facciamo/malattie-senza-diagnosi), through which each patient will be carefully evaluated for WES testing.

**References**

1. Sawyer SL, Hartley T, Dyment DA et al. Utility of whole-exome sequencing for those near the end of the diagnostic odyssey: time to address gaps in care. Clin Genet. 2016;89:275-84.

2. Valencia CA, Husami A, Holle J et al. Clinical impact and cost-effectiveness of whole-exome sequencing as a diagnostic tool: a pediatric center’s experience. Front Pediatr. 2015;3:67.

3. Shashi V, McConckie-Rosell A, Rosell B et al. The utility of the traditional medical genetics diagnostic evaluation in the context of next-generation sequencing for undiagnosed genetic disorders. Genet Med. 2014;16:176-82.

## A28 Ivacaftor treatment in cystic fibrosis and improvement in resting energy expenditure, gut inflammation, and fat absorption

### Virginia A Stallings^1,2^, Chiara Berlolaso^3^, Carolyn McAnlis^1^, Joan I Schall^1^

#### ^1^Gastroenterology, Hepatology and Nutrition, Children’s Hospital of Philadelphia, Philadelphia, PA 19104, USA; ^2^ Perelman School of Medicine, University of Pennsylvania, Pediatrics, Philadelphia, PA 19104, USA; ^3^ Department of Pediatrics, Sapienza University, 00161 Rome, Italy

##### **Correspondence:** Virginia A Stallings (stallingsv@email.chop.edu)

**Background**

Ivacaftor is a therapy for people with cystic fibrosis transmembrane conductance regulator (CFTR) gating mutations, which results in weight gain and improved pulmonary function. The mechanisms of the weight gain have not been determined.

**Materials and methods**

This was an observational study of children and adults (≥5 yrs old) from North America and Italy with one or more CFTR gating mutations before and after 3-month Ivacaftor treatment. Height, weight, and BMI were measured. Fat mass (FM) and fat free mass (FFM) were assessed by whole body dual x-ray absorptiometry (DXA). Forced expiratory volume at one second percent predicted (FEV1%) was assessed by spirometry, total energy expenditure (TEE) by doubly labeled water method, resting energy expenditure by indirect calorimetry (expressed % predicted using Schofield [REE%]), and coefficient of fat absorption (CFA) from 72-hour stool and 3-day weighed food records. Fecal calprotectin (μg/g stool) was assessed for gut inflammation and fecal elastase (μg/g stool) for pancreatic status. All study visits were conducted at the Children’s Hospital of Philadelphia.

**Results**

Twenty-three subjects (5 to 61 yr, mean age 17.3±13.0 yr) from the USA, Canada and Italy completed the study: 74% were pancreatic insufficient (PI) and receiving pancreatic enzyme medication. After 3-months of Ivacaftor treatment, weight gain of 2.5±2.2 kg (44.1±16.0 to 46.6±16.2 kgs) and improvement in FEV1% of 10±12% (86±21 to 95±20%) were significant (p<0.001). Both FFM (0.9±1.9 kg) and FM (1.6±1.5 kg) components increased (p<0.05). REE% declined 5.5±12.0% (95.6±11.2 to 90.1±10.3%, p<0.05) as did fecal calprotectin 30±40 μg/g stool (78±116 to 47±108 μg/g/stool, p<0.01). TEE remained stable with final results pending. Changes were greater in subjects with PI, and CFA improved 3.1±3.0% in this group. The change (∆) in weight was positively correlated with ∆FEV1% (r=0.46, p=0.028) and ∆CFA (r=0.47, p=0.032) and negatively with ∆REE% (r=-0.50, p=0.017). ∆FEV1% was negatively correlated with ∆REE% (r=-0.64, p=0.001), and ∆calprotectin (r=-0.49, p=0.022).

**Conclusion**

3-month Ivacaftor treatment resulted in gain of FM and FFM and improved pulmonary function in people with CFTR gating mutations. This improvement was associated with decline in REE and reduced gut inflammation and fat malabsorption, and was greater for those with CF and PI.

**Trial registration.** ClinicalTrials.gov (NCT02141464)

**Funding**

Supported by Vertex Pharmaceutical Inc., Clinical Translational Research Center (UL1RR024134 & UL1TR000003), Nutrition Center at Children’s Hospital of Philadelphia.

## A29 Prolonged acute convulsive seizure/status epilecticus: a proposal of agreed treatment plan

### Pasquale Striano (pasqualestriano@ospedale-gaslini.ge.it)

#### Department of Neurosciences, Rehabilitation, Ophthalmology, Genetics, Maternal and Child Health, University of Genova, Genoa, Italy

Status Epilepticus (SE) is the most common neurological emergency of childhood, with an incidence between 17 and 23 per 100000 children per year. [1] The management of children at risk of prolonged, acute convulsive seizures outside of the hospital is a poorly-researched subject in the field of epilepsy. In Italy and elsewhere, clinical guidelines offer little guidance on how these seizures should be managed outside of the hospital. Comprehensive protocols, which provide a clear framework for action and a seamless pathway of care for children from the time they are prescribed rescue medication to all potential situations where this medication may need to be administered, are needed. Clinically, SE is divided into four subsequent stages: early, established, refractory, and super-refractory. [2] During the prodromal or incipient stage (<5 minutes) it is unknown whether the seizure will self-terminate or evolve into SE. Persisting SE has been divided into early SE (5 to 10 minutes), established SE (10 to 30 minutes), refractory SE (RSE) (30 to 60 minutes or seizures that persist despite treatment with adequate doses of two or three anticonvulsants) or super-refractory SE (>24 hours or seizures that continue despite treatment with anaesthetics). [3] To provide guidance for the acute treatment of SE, we propose an expert practice guideline. The need for early pharmacologic intervention stresses the need for action in the prehospital setting, generally using benzodiazepines. When first-line drugs fail, levetiracetam or sodium phenytoin should be generally used. In cases of refractory SE, pharmacologic options can be continuous intravenous infusion drugs to suppress electroencephalographic bursts and convulsive activity.

**References**

1. Abend N, Loddenkemper T. Pediatric Status Epilepticus management. Current Opinion Pediatrics. 2014;26:668-674.

2. Trinka E, Höfler J, Leitinger M. Pharmacotherapy for Status Epilepticus. Drugs. 2015; 75:1499–1521.

3. Brophy GM, Bell R, Claassen J et al. Guidelines for the evaluation and management of status epilepticus, Neurocrit Care. 2012;17:3-23.

## A30 The obese teenager and compliance to diets

### Rita Tanas^1^, Giulia De Iaco^2^, Maria Marsella^3^, Guido Caggese^4^

#### ^1^UO Pediatria Az Ospedaliero Universitaria, Cona, Ferrara, Italy; ^2^Centro per il trattamento dei Disturbi del Comportamento Alimentare, Todi, Perugia, Italy; ^3^UOC di Pediatria, Az. Ospedaliera di Rilievo Nazionale “San G.Moscati”, Avellino, Italy; ^4^Formazione Professionale, Azienda Ospedaliero Universitaria, Ferrara, Italy

##### **Correspondence:** Rita Tanas (tanas.rita@tin.it)

In adolescence compliance for pharmacological therapies in chronic diseases turns out to be only 45%, for dietary lifestyle 33%; only 5% of families adhere to the mediterranean diet [1-3]. Compliance to dietotherapy is more demanding: it takes 24 hours a day and the results are disappointing [4-6].

Obese teenagers suffer for weight stigma and bullism which is continuously increasing in all fields [7], so they have difficulty complying with projects of cure [8].

The “compliance with diet” is a challenge. Very few paediatricians and other caregivers feel courageous enough to accept it and many refuse to still hear about obesity, especially in adolescence.

However, everywhere teenagers ask to be helped with these problems [9-10]. So what then?

If we want to be helpful we have to listen to teenagers, finding a successful course focused on them, searching for the causes of their failure and new ways of improvement [11].

**Fig. 2 (abstract A30).**
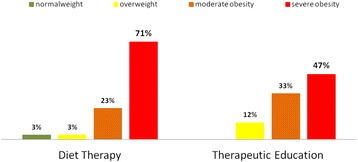
Study on 88 adolescents with severe obesity (BMI >99°pc WHO), median age 12 ± 2 yrs, F 45%, of which 57 treated with Therapeutic Education and 31 with Dietotherapy. At T0: BMI 30.2 ± 5, BMI z score 2.98 ± 0,4. Grade of excess weight after 2.5 ± 1.3 years in the two groups. [Tanas R Congresso Nazionale Società Italiana di Medicina dell’Adolescenza, Palermo 2012]

**Fig. 3 (abstract A30).**
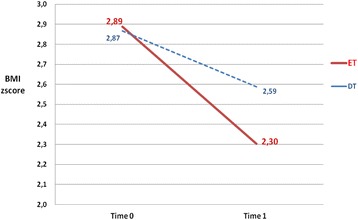
Study on 88 adolescents with severe obesity (BMI >99°pc WHO), median age 12 ± 2 yrs, F 45%, of which 57 treated with Education Therapy and 31 with Dietotherapy. At T0: BMI 30.2 ± 5, BMI z score 2.98 ± 0,4. Change in BMI z score after 2.5 ± 1.3 years in the two groups. [Tanas R Congresso Nazionale SIMA Palermo 2012. ET Education Therapy, DT Dietotherapy]

Twenty years ago I started a project of therapeutic family education: “Perle e Delfini” [12-14] obtaining good results also with adolescents with severe obesity after 2,5 years of follow-up (Figs. 2 and 3). Because of the risk of developing eating disorders, I gave up on the prescription of “strict diets” [15]. I realized that only avoiding diets and food diaries, I could convey a message of trust. The program replaces the prescribed diet with a family education on the importance of a healthy life style, without prohibitions, explaining the importance of portions and caloric density, and supporting self-efficacy (i.e. belief in success) with the therapeutic narration. Other authors supported successfully similar programs [16] and nowadays reduction and adaptation of objectives are promoted [17-18]. A therapeutic project has to focus on behaviors and not on kilograms to be lost, and it has to reward the achievements of psycho-physical health. The long-lasting change of behaviors can be supported only with self-efficacy, while prescribing a diet, also slightly low calories and rich of alternatives, is a message of mistrust: like “breaking someones leg and then asking him/her to run”. We have to heal the legs that derision in family, school and health setting [19-21] has broken. We have to become aware of our professional weight stigma and start working with them, to have teenagers less obese and more healthy, without dangerous adherence to diets.

**References**

1. Advisory Board sull’Aderenza, Bartolini F, Caputi AP, et al. Manifesto per l’Aderenza alla terapia farmacologica sul territorio italiano. Gennaio 2013. Accessed at http://www.diabeteitalia.it/download.aspx?sfn=3d4c0679-2a9f-4114-85f8-06cf16fcd6a9.

2. Speri L, Brunelli M. Genitori più Prendiamoci più cura della loro vita 7 azioni per la vita del tuo bambino. Materiale informativo per operatori. Edizione Cierre Grafica. Verona 2009. Accessed at http://www.salute.gov.it/imgs/C_17_opuscoliPoster_126_allegato.pdf

3. Censi L, D’Addesa D, Galeone D, Andreozzi S, Spinelli A (Ed.). Studio ZOOM8: l’alimentazione e l’attività fisica dei bambini della scuola primaria. Roma: Istituto Superiore di Sanità; 2012.

4. Clifton PM. Dietary treatment for obesity. Nat Clin Pract Gastroenterol Hepatol. 2008;5:672-81.

5. Mann T, Tomiyama AJ, Westling E et al. Medicare's search for effective obesity treatments: diets are not the answer. Am Psychol. 2007;62:220-33.

6. Wadden TA, Stunkard AJ (Ed.). Handbook of obesity treatment. New York: The Guilford Press; 2002.

7. Andreyeva T, Puhl RM, Brownell KD. Changes in perceived weight discrimination among Americans 1995-1996 through 2004-2006. Obesity (Silver Spring). 2008;16:1129-34.

8. Puhl RM, Peterson JL, Luedicke J. Weight-based victimization: bullying experiences of weight loss treatment-seeking youth. Pediatrics. 2013;131:e1-9.

9. Viner RM. Adolescents' health needs: the same the world over. Arch Dis Child. 2013;98:2.

10. Rees RW, Caird J, Dickson K et al. 'It's on your conscience all the time': a systematic review of qualitative studies examining views on obesity among young people aged 12-18 years in the UK. BMJ Open. 2014;4:e004404.

11. Dietz WH, Baur LA, Hall K et al. Management of obesity: improvement of health-care training and systems for prevention and care. Lancet. 2015;385:2521-33.

12. Tanas R, Marcolongo R, Pedretti S et al. A family-based education program for obesity: a three-year study. BMC Pediatr. 2007;7:33.

13. Tanas R, Marcolongo R. Una cura senza dieta per l’adolescente soprappeso: l’educazione terapeutica. Rivista Italiana di Medicina dell’Adolescenza. 2007;5:17-24.

14. Tanas R, Pedretti S, Greggio MS. L’obesità in età adolescenziale: problemi aperti. La comunicazione della diagnosi e la motivazione alla cura. Rivista Italiana di Medicina dell’Adolescenza. 2005;3 (Suppl.2):116.

15. Vartanian LR, Porter AM. Weight stigma and eating behavior: A review of the literature. Appetite. 2016;102:3-14.

16. Savoye M, Shaw M, Dziura J et al. Effects of a weight management program on body composition and metabolic parameters in overweight children: A randomized controlled trial. JAMA. 2007; 24: 2697-2704.

17. Holm JC, Nowicka P, Farpour-Lambert NJ et al. The ethics of childhood obesity treatment - from the Childhood Obesity Task Force (COTF) of European Association for the Study of Obesity (EASO). Obes Facts. 2014;7:274-81.

18. Ross R, Blair S, de Lannoy L et al. Changing the endpoints for determining effective obesity management. Prog Cardiovasc Dis. 2015;57:330-6.

19. Ruffman T, O'Brien KS, Taumoepeau M et al. Toddlers' bias to look at average versus obese figures relates to maternal anti-fat prejudice. J Exp Child Psychol. 2016;142:195-202.

20. Musher-Eizenman DR, Holub SC, Hauser JC et al. The relationship between parents' anti-fat attitudes and restrictive feeding. Obesity (Silver Spring). 2007;15:2095-102.

21. Jendrzyca A, Warschburger P. Weight stigma and eating behaviours in elementary school children: A prospective population-based study. Appetite. 2016;1;102:51-9.

## A31 Therapeutic Diagnostic appropriateness: imaging

### Paolo Toma (paolo.toma@opbg.net)

#### Department of imaging, Bambino Gesù Hospital IRCCS, 00165 Rome, Italy

Rules of evidence-based medicine were adopted by radiology later than by clinical specialties. While interventions efficacy is typically proved by randomized trials, the practice of imaging is often founded on lower levels of evidence. Only recently, authors of systematic reviews began to assess accuracy of imaging modalities [1].

Moreover it was proven that only 38% (330 of 867) of systematic reviews on radiology published from January 2001 to December 2010 included imaging specialists as authors (first author in 176 (20%)). Only 26% were published in imaging journals [2].

Very often the biases are in the first steps of the study: Was the equipment adequately updated? Were technical factors, sections, mathematical reconstructions methods correct? Was the knowledge of those who decoded the data adequate?” [3].

Unfortunately there is another source of bias: is the experience of peer reviewers of clinical journals sufficient?

The other problem is that interests of medical industries increasingly setting the research agenda can mislead the evidence of diagnostic efficacy [4].

Practically, in "Screening Radiology" appropriateness is closely linked to political and economic choices. These affect less the "Clinical Radiology". However, the two fields are closely related and the equilibrium is difficult to be mantained.

As a rule, in the study of symptomatic patients (Clinical Radiology) we’ll try to use high-sensitivity investigations and we’ll accept a low specificity that can be balanced by further diagnostic procedures.

Conversely, in the study of asymptomatic subjects (screening Radiology) we’ll try to use investigations with high specificity, accepting also the price of a lower sensitivity.

We know that accuracy measures can be correlated: in a setting where sensitivity is higher, specificity tends to be lower, and vice versa.

**References**

1. McInnes MD, Bossuyt PM. Pitfalls of Systematic Reviews and Meta-Analyses in Imaging Research. Radiology. 2015;277:13-21.

2. Sardanelli F, Bashir H, Berzaczy D, et al. The role of imaging specialists as authors of systematic reviews on diagnostic and interventional imaging and its impact on scientific quality: report from the EuroAIM Evidence-based Radiology Working Group. Radiology. 2014;272:533-40.

3. Di Leo G, Sardanelli F. Pitfalls of Systematic Reviews and Meta-Analyses. Radiology. 2016;279(2):652.

4. Greenhalgh T, Howick J, Maskrey N; Evidence Based Medicine Renaissance Group. Evidence based medicine: a movement in crisis? BMJ. 2014;348:g3725.

## A32 Migrant children in Italy: migratory history and health profile, infective aspects

### Piero Valentini^1^, Danilo Buonsenso^2^, David Pata^1^, Manuela Ceccarelli^3^

#### ^1^Department of Health Sciences of the Woman and Child, Pediatrics, "A. Gemelli " Foundation University Hospital, Rome, Italy; ^2^DEA, Pediatric Emergency, Pediatric Hospital “Bambino Gesù”, I.R.C.C.S., Rome, Italy; ^3^Department of Specialistic Medicine, Infectious Diseases Specialization School, University of Messina, Messina, Italy

##### **Correspondence:** Piero Valentini (piero.valentini@policlinicogemelli.it)

Despite what you might think, the migrant status does not systematically associate with infectious diseases [1], nor particularly rare diseases. Infectious diseases, however, although can indiscriminately strike individuals from all social categories, are indeed an issue directly linked to poverty and this aspect most commonly affects people forced to emigrate by economic necessity or by wars. The migrant children are divided in different categories: recently immigrated, with families or alone, refugees, adopted, born in our country from immigrated parents. The probability to have infectious diseases and the approach for their detection have different aspects and problems. Immigrants and refugees are most directly at risk of infectious diseases because of migration process, often long and in extremely difficult conditions, which expose them to all those conditions (crowding, poor hygiene, lack of medical care) that can easily lead to epidemic situations, characterized primarily by respiratory and gastrointestinal diseases. Special consideration has to be given to infections transmitted by arthropods, which may occur even a long time after the arrival of the child in Italy: in a cohort of children newly arrived from the Democratic Republic of Congo many cases of malaria were observed, and biomolecular techniques proved helpful in order to identify the forms of P. ovale, cause of relapses. Children internationally adopted are at lower risk of acute illness, but may show infectious diseases related to epidemiologic situation of their countries, the care they had in the pre-adoption period and a vaccination immunity often uncertain, in spite of the accompanying documentation: 408 out of 902 (45,23%) children evaluated at December 31, 2015, had infectious diseases of various kinds and / or vaccination coverage deficit (personal data). Children born in our country from immigrated parents have a particular risk associated with adults diseases: tuberculosis, that is not endemic in Italy, is currently observed in immigrants with a higher prevalence than natives and this epidemiological data persists in children belonging to these families [2]. In a recent multicenter work [3], out of 2339 immigrated or adopted children, 60.4% was found to be suffering from latent tuberculosis infection (LTBI) and 5.6% from active tuberculosis. Therefore, beyond special situations, as reception or sorting centers, where it is good to implement control systems to identify quickly potential risk of epidemic, hospital and family pediatricians meeting migrant children must be aware of the migration trajectory, the country of origin, recent trips to the native land and family economic and logistic situation, to speculate and detect quickly and appropriately diseases that can affect preferentially these categories of children.

**References**

1. WHO. Migration and health: key issues. Accessed at: http://www.euro.who.int/en/health-topics/health-determinants/migration-and-health/migrant-health-in-the-european-region/migration-and-health-key-issues (04/11/2016)

2. Buonsenso D, Lancella L, Delogu G, et al. A twenty-year retrospective study of pediatric tuberculosis in two tertiary hospitals in Rome. Pediatr Infect Dis J. 2012;31:1022-26.

3 Galli L, Lancella L, Tersigni C, et al. Pediatric tuberculosis in italian children: epidemiological and clinical data from the italian register of pediatric tuberculosis. Int J Mol Sci. 2016, 17, 960.

## A33 Nutritional challenges in adolescence

### Elvira Verduci, Marta Brambilla, Benedetta Mariani, Carlotta Lassandro, Alice Re Dionigi, Sara Vizzuso, Giuseppe Banderali

#### Department of Health Sciences, San Paolo Hospital, University of Milan, Milan, Italy

##### **Correspondence:** Elvira Verduci (elvira.verduci@unimi.it)

Improving nutrition is a key opportunity to improve health. Adolescence is a critical period: many important physical and psychologic changes occur in a very short period. Adolescence has to be considered an especially nutritionally vulnerable period: first, there is a greater demand for nutrients because of the dramatic growth and development, second there are many changes in lifestyle and food habits [1]. While the energy intake is higher during adolescence, due to the intense needs for growth (4-5% of total daily energy needs), the macronutrient intake is unchanged (carbohydrates: 45-60%; lipids: 20-35%; proteins: 12-15%) [2]. Adolescence is characterized by undesiderable changes in eating behaviours: increased consumption of sugar sweetened beverages, calorie-dense, nutrient poor snacks and a decline in the consumption of milk, fruits and vegetables. Meals patterns tend to change: teenagers are more likely to skip breakfast, less likely to participate in family dinners and frequently eat away from home (e.g. fast foods) [3].

Changes in dietary habits towards an unbalanced diet could induce nutrition-related disorders, both qualitative (e.g. micronutrient deficiencies) and quantitative (e.g. obesity and its comorbidities) [4]. Since nutritional habits established during adolescence are likely to track into adulthood, specific action are needed to improve the quality of the diet of adolescences [5]. The HELENA study identified deficient concentrations for plasma folate, vitamin D, vitamin B-6, β-carotene and vitamin E. Vitamin D and folate are the vitamins most at risk [4]. Vitamin B6, folate, and vitamin B12 deficiencies are considered as a risk factor in cardiovascular diseases, neural tube defects and some types of cancers. They are involved in optimal cognitive function and bone health. Sub-clinical deficiencies of vitamin B6, folate, and vitamin B12 status are not uncommon during adolescence [6]. Vitamin D status is a key determinant of bone health during childhood and adolescence. Vitamin D deficiency or insufficiency may negatively affect bone mineralization: adequate muscle mass accrual is essential for the attainment of peak bone mass [7]. Iron has a role in prefrontal dopamine signalling and could be involved in impaired executive functioning, which includes planning, working memory, self-monitoring and regulation, inhibition and volition. Mild iron deficiency, which is common in menstruating adolescent girls, has implications in well-being and so should be recognised and treated [8].

In conclusion, public authorities should raise awareness of the importance of nutrition in critical periods of life, such as adolescence.

**References**

1. Spear BA. Adolescent growth and development. J Am Diet Assoc. 2002;102:S23-29.

2. SINU (Italian Society for Human Nutrition). LARN- Intake Levels of Reference for Nutrients and Energy). IV Revision. SINU-INRAN. Milano: SICS, 2014.

3. Birch L, Savage JS, Ventura A. Influences on the development of children’s eating behaviours: from infancy to adolescence. Can J Diet Pract Res. 2007;68:s1-s56.

4. Moreno LA, Gottrand F, Huybrechts I, et al. Nutrition and lifestyle in European adolescents: the HELENA (Health Lifestyle in Europe by Nutrition in Adolescence) Study. Adv. Nutr. 2014;5:615S-623S.

5. Branca F, Piwoz E, Schultink W, Sullivan LM. Nutrition and health in women, children, and adolescent girls. BMJ. 2015; 351:h4173.

6. Iglesia I, Mouratidou T, González-Gross M, et al. Foods contributing to vitamin B6, folate, and vitamin B12 intakes and biomarkers status in European adolescents: the HELENA study. Eur J Nutr. 2016. Epub ahead of print.

7. Saggese G, Vierucci F, Boot AM, et al. Vitamin D in childhood and adolescence: an expert position paper. Eur J Pediatr. 2015; 174:565-576.

8. Scott SP, Murray-Kolb LE. Iron status is associated with performance on executive functioning tasks in nonanemic young women. J Nutr. 2016; 146: 30-37.

## A34 Neurological complications in thyroid diseases: neurological profile

### Gianvito Panzarino, Claudia Di Paolantonio, Alberto Verrotti

#### Department of Pediatrics, University of L’Aquila, L'Aquila, Italy

Thyroid hormones exert critical roles for brain development, in particular influencing various aspects of the neuronal development.

In prenatal and postnatal life, deficiency of thyroid hormones can have a detrimental effect on cerebral maturation with subsequent neuromotor impairment. Hypothyroidism (both in mother and/or newborn) associated or not to iodine deficiency is the main cause of this situation [1]. Moreover mother’s hypothyroidism can determine an high probabilities of developing autism spectrum disorders [2].

Among the most frequent treatable conditions of psychomotor impairment, we must always consider congenital hypothyroidism (CH). The situation can be permanent (abnormality gland development or defect hormonogenesis), less commonly is transient.

The clinical manifestations of CH are subclinical, therefore some newborns can be underdiagnosed and the retarded diagnosis can cause severe consequences [3]. Substitutive therapy must be started early in order to reach euthyroidism as soon as possible. A possible inverse correlation between mental development and the beginning of therapy has been suggested. Thyroid hormone treatment is recommended as T4: 10-15 μgm/kg/day, in order to obtain T4 and TSH normal values [3]. Wheeler et al described adolescents with CH and abnormal hippocampal functioning [4].

The introduction of neonatal screening is an important tool to prevent severe cognitive consequences.

Hashimoto thyroiditis and Graves' disease can be associated with many neurological disturbances [5]. Patients with Hashimoto encephalopathy (HE) can display neuromuscular disturbance and epilepsy with abnormalities of white matter (found in brain magnetic resonance imaging). In the majority of cases the diagnosis is based on the presence of elevated thyroid antibodies. In HE patients high protein concentrations are often present in the cerebrospinal fluid. The first-line therapy is based on the use of steroids, alternative therapy include IV immunoglobulin and plasmapheresis [6].

References

1. Bernal J. Thyroid Hormones in Brain Development and Function. De Groot LJ, Beck-Peccoz P, Chrousos G, et al., editors. Endotext. 2000.

2. Berbel P, Navarro D, Roman GC. An Evo-Devo Approach to Thyroid Hormones in Cerebral and Cerebellar Cortical Development: Etiological Implications for Autism. Front Endocrinol. 2014; 5: 146.

3. Rose SR, Brown RS. Update of Newborn Screening and Therapy for congenital Hypothyroidism. Pediatrics. 2006; 117:2290-2303.

4. Wheeler SM, McLelland VC, Sheard E, McAndrews MP, Rovet JF. Hippocampal Functioning and Verbal Associative Memory in Adolescents with Congenital Hypothyroidism. Front Endocrinol. 2015; 6:163.

5. Nandi-Munshi D, Taplin CE. Thyroid-related neurological disorders and complications in children. Pediatr Neurol. 2015; 52:373-382.

6. Zhou JY, Xu B, Lopes J, Li L. Hashimoto encephalopathy: literature review. Acta Neurol Scand. 2016 In press.

## A35 Anti-meningococcal prevention in pediatrics

### Alberto Villani, Elena Bozzola, Laura Cursi, Annalisa Grandin, Andrzej Krzysztofiak, Laura Lancella

#### General Pediatrics and Infectious Disease – Bambino Gesù Children Hospital – Rome - Italy

Invasive meningococcal disease (IMD) is a severe problem all over the world. The individuation of children with IMD is important to promptly prescribe systemic antibiotic treatment and to try avoiding death and/or invalidating sequelae.

Nevertheless, vaccination remains the best strategy to prevent meningococcal disease. ^1^ In fact, in order to eliminate an infectious disease, the first essential requirement is undoubtedly the availability of both safe and effective vaccines and a strategic planning of immunization.^2^

There are 12 known serogroups of Neisseria meningitidis, as determinated by antigens of the Neisseria meningitidis polysaccharide capsule. A, B, C, W, X, and Y are the serogroups which may cause invasive human disease.

Serogroups A, C, Y, and W may be prevented with immunization by a quadrivalent capsular polysaccharide-protein conjugate vaccine. Conjugate vaccines against Neisseria Meningitidis C has dramatically reduced cases of bacterial meningitis in the industrialized nations, also in Italy. Meningitis type B continues to be a threat to children and adolescents in Italy and worldwide. Serogroup B is actually known as the most common cause of IMD. The immunization is recently available through a recombinant protein vaccine. ^3^ In order to eliminate an infectious disease, the first essential requirement is undoubtedly the availability of safe and effective vaccines. However, strategic planning of the application of the vaccines which have become available is equally important.

References

1. Bosis S., Mayer A., Esposito S. Meningococcal disease in childhood: epidemiology, clinical features and prevention. J Prev Med Hyg 2015; 56: E121-E124.

2. Gasparini R., Amicizia D., Lai P.L., Panatto D. Meningococcal B vaccination strategies and their practical application in Italy. J Prev Med Hyg 2015; 56: E133-E139.

3. Baker C. J. Prevention of Meningococcal Infection in the United States: Current Recommendations and Future Considerations. J Adol Health 2016; 59: S29-S37.

## A36 Unaccompanied foreigner children (minors) in Italy: holistic multidisciplinary protocol for age assessment

### Raffaele Virdis^1,2,4^, Patrizia Carletti^1,3^

#### ^1^Board “Immigrants and Health Services” of the Health Commission of The Italian Regions Conference, Rome, Italy; ^2^Consultant, GLNBM, Florence, Italy; ^3^Coordinator of the National Board. Observatory on Health Inequalities, Health Department, Marche Region, Ancona, Italy; ^4^Formerly of Parma University, Parma, Italy

##### **Correspondence:** Raffaele Virdis (raffaele.virdis@unipr.it)

**Background**

Between 2012 and 2016, the Technical Interregional Board “Immigrants and Health Services” of the Health Commission of The Italian Regions Conference, together with the contribution of various Ministries, Scientific Societies, and stakeholders (UNHCR, Save the Children), set up a “Protocol for the identification and the holistic multidisciplinary age assessment of foreigner, unaccompanied children”. The purposes were to create a protocol coherent with the European directives [1] and obtain clear and feasible indications for age assessment (AA) in these minors, passing the criticalities due to diverse methods, procedures and judgment parameters adopted in the different Italian Regions, which were often based on a single assessment using invasive tests (mainly radiological determination of bone or dental age). These procedures were considered affecting human rights (according to International law courts’ opinions) when used for lawful purposes, while the scientific literature underlined the frequent inaccuracy [2, 3] .

**Methods**

The experts of the “Board” have underlined the central role of the pediatrician of the “National Health Service” in this AA procedure, but they have, also, pointed out that the physical examination (pediatric and auxological examination, eventual instrumental tests) used as the only tool, is aleatory because relative to the degree of body and pubertal maturation and not always to the chronological age.

The AA performed through medical methods could not give an exact response and, if in the 95% of the cases the possible error is ± 2 years, in the remaining 5% could be also ± 3-4 years. This range of variability is not acceptable, especially in ages near the legal limit of 18 years.

This proposed holistic and multidisciplinary procedure takes into account not only the body but also the psychological and behavioral maturation (child neuropsychiatrist and/or psychologist) and the social and cultural situation (social worker and cultural mediator). Moreover, this method guarantees to the alleged minor the respect for his person and his legal protection, and it seems to be the best system, even if the result could be not completely correct because any form of AA is not an “exact science”.

**Conclusions**

The protocol in comparison with other international ones and with what was done before, confirms its scientific nature and the respect for human rights, providing recourse to invasive tests only in extreme situations and always with the informed consent of the subject. All these aspects and procedures involve economic costs, time, training and continuous updating of the professional figures.

**References**

1. Directive 2013/32/ of the European Parliament and of the Council of 26 June 2013 and Directive 2013/33 of 26 June 2013

2. Aynsley-Green A., Cole T.J., Crawley H., Lessof N., Boag L.R., Wallace R.M.: Medical, statistical, ethical and human rights considerations in the assessment of age in children and young people subject to immigration control. Br Med Bulletin. 2012;102:17-42

3. Benso L., Milani S. Alcune considerazioni sull’uso forense dell’età biologica, 12 giugno 2013. Accessed at http://www.asgi.it/wp-content/uploads/public/1_2013_accertamento_eta_materiali.pdf (04/11/2016)

## A37 Neurological complications during thyroid diseases, thyroid dysfunctions: clinical scenarios in children

### Giovanna Weber, Silvana Caiulo, Maria Cristina Vigone

#### Vita-Salute San Raffaele University, Department of Pediatrics, IRCCS San Raffaele Hospital, Milan, Italy

##### **Correspondence:** Giovanna Weber (weber.giovanna@hsr.it)

Thyroid hormones are crucial for an adequate neuropsychological development in the first years of life. During pregnancy, the fetus is susceptible to not treated maternal hypothyroidism. A study published on NEJM [1] showed that the full-scale intellective quotient scores of children born from mothers with thyroid deficiency averaged 7 points lower than those of the matched control children. Subsequent studies [2,3] have confirmed that subclinical hypothyroidism and hypothyroxinemia during pregnancy might led to altered neuropsychological development in the child. Therefore, in pregnancies with risk factors for thyroid dysfunction international guidelines have been delineated in order to evaluate maternal thyroid function precociously and to keep TSH and FT4 values in the correct reference ranges for pregnancies [4].

Congenital hypothyroidism (CH) is a frequent preventable cause of mental retardation. The eradication of mental retardation caused by CH has been possible thanks to the realization of neonatal screening programmes for CH, a precocious diagnosis (before 15 days of life) and an optimised initial therapeutic dosage of levothyroxine (10-15 mcg/kg/day). Studies published in the last 10 years have shown an average intellective quotient 20 points higher compared to pre-screening era. However, in a minority of patients, there might be minimal neurological deficits (minor deficits in psychomotricity, concentration, attention, and a delayed acquisition of language), despite normal intellective quotient. Risk factors for intellectual disability that should be taken into consideration are CH severity, the adequacy of treatment, and the socio-educational status of the parents [5]. Even an excessive dose of levothyroxine should be avoided for possible attention deficits [6].

With early and adequate treatment, intellectual disability caused by CH has largely become a thing of the past. However, there are exceptional cases of hypothyroidism due to genetic mutations that are associated to severe neurological impairment, such as the Allan-Herndon-Dudley syndrome and the brain-lung-thyroid syndrome. Allan-Herndon-Dudley syndrome is caused by mutations in the SLC16A2 gene, also known as MCT8. It is a rare disorder of brain development that occurs exclusively in males and causes moderate to severe intellectual disability, hypotonia, spasticity, distonia and epilepsy. Brain-lung-thyroid syndrome is characterized by CH or subclinical hypothyroidism, infant respiratory distress syndrome and benign hereditary chorea.

**References**

1. Haddow JE, Palomaki GE, Allan WC, et al. Maternal thyroid deficiency during pregnancy and subsequent neuropsychological development of the child. N Engl J Med. 1999;341:549-55.

2. Henrichs J, Bongers-Schokking JJ, Schenk JJ, et al. Maternal thyroid function during early pregnancy and cognitive functioning in early childhood: the generation R study. J Clin Endocrinol Metab. 2010;95:4227-4234.

3. Li Y, Shan Z, Teng W, . et al. Abnormalities of maternal thyroid function during pregnancy affect neuropsychological development of their children at 25-30 months. Clinical Endocrinol. 2010; 72, 825–829.

4. Stagnaro-Green A, Abalovich M, Alexander E,. et al. American Thyroid Association Taskforce on Thyroid Disease During Pregnancy and Postpartum.Guidelines of the American Thyroid Association for the diagnosis and management of thyroid disease during pregnancy and postpartum. Thyroid. 2011;21:1081-125.

5. Léger J. Congenital hypothyroidism: a clinical update of long-term outcome in young adults. Eur J Endocrinol. 2015;172, R67–R77.

6. Rovet J, Alvarez M. Thyroid hormone and attention in school-age children with congenital hypothyroidism. J Child Psychol Psychiatry. 1996;37:579–585

